# Loss of CSE couples with BRAF V600E to fuel thyroid cancer metastatic progression through zinc-dependent activation of the EMT machinery

**DOI:** 10.1186/s13046-026-03752-0

**Published:** 2026-06-10

**Authors:** Shichen Xu, Huixin Yu, Hongxun Wu, Zongfu Pan, Jiajie Xu, Yun Zhu, Botao Huang, Gangming Cai, Xiaobo Gu, Min Yang, Donghui Pan, Jie Pan, Jing Wu, Xian Cheng, Jiandong Bao, Li Zhang

**Affiliations:** 1 National Health Commission Key Laboratory of Nuclear Medicine, Jiangsu Key Laboratory of Molecular Nuclear Medicine, Jiangsu Institute of Nuclear Medicine, 20 Qian Rong Road, Wuxi, 214063 China; 2 Jiangsu Institute of Nuclear Medicine Jiangyuan Hospital of Jiangsu Province, Wuxi, 214063 China; 3 Department of Pharmacy, Center for Clinical Pharmacy, Cancer Center, Zhejiang Provincial People’s Hospital (Affiliated People’s Hospital), Hangzhou Medical College, Hangzhou, 310014 China; 4 Department of Head and Neck Surgery, Otolaryngology & Head and Neck Center, Cancer Center, Zhejiang Provincial People’s Hospital, Hangzhou, China; 5 Department of Radiopharmaceuticals, School of Pharmacy, Nanjing Medical University, Nanjing, 211166 China; 6 School of Life Sciences, Nanjing Medical University, Changzhou, China; 7 School of Life science and Technology, Southeast University, Nanjing, 210096 China

**Keywords:** Papillary thyroid carcinoma, BRAF V600E, Metastasis, cystathionine γ-lyase, hydrogen sulfide, Zinc homeostasis

## Abstract

**Background:**

BRAF V600E, the principal oncogenic driver in papillary thyroid carcinoma (PTC), strongly correlated with lymph node metastasis, yet the underlying molecular mechanisms remain elusive.

**Methods:**

This study screened effector related to the BRAF V600E mutation and PTC progression through bioinformatics analysis. The precise functional roles and regulatory networks of cystathionine γ-lyase (CSE), a primary hydrogen sulfide (H₂S)-producing enzyme, in thyroid cancer metastasis were then investigated in cell and mice models. Finally, these findings were further validated in a cohort of clinical pathological samples.

**Results:**

We identified that CSE is aberrantly silenced in advanced thyroid cancer. Mechanistically, BRAF V600E-driven MAPK activation upregulates *miR-31-5p*, which directly targets the *CSE* 3’UTR to inhibit its translation. The consequent H₂S deficiency disrupts intracellular metallo-homeostasis, triggering intracellular zinc accumulation that stabilizes Zeb1, MMP-2, thereby orchestrating the epithelial-mesenchymal transition (EMT). Critically, the metastatic potential of thyroid cancer cells relies strictly on CSE enzymatic activity. Pharmacological reconstitution of the H₂S pool using exogenous donors effectively bypasses CSE enzymatic deficiency, eliminates excess zinc, and reactivates metastasis-suppressive signaling.

**Conclusions:**

These findings uncover a novel *miR-31-5p*/CSE/H₂S/Zinc axis that fuels BRAF-driven progression. These findings provide a compelling mechanistic rationale for utilizing H₂S-based interventions as a potential therapeutic strategy against BRAF-driven thyroid cancer metastasis.

**Graphical Abstract:**

**Figure:**
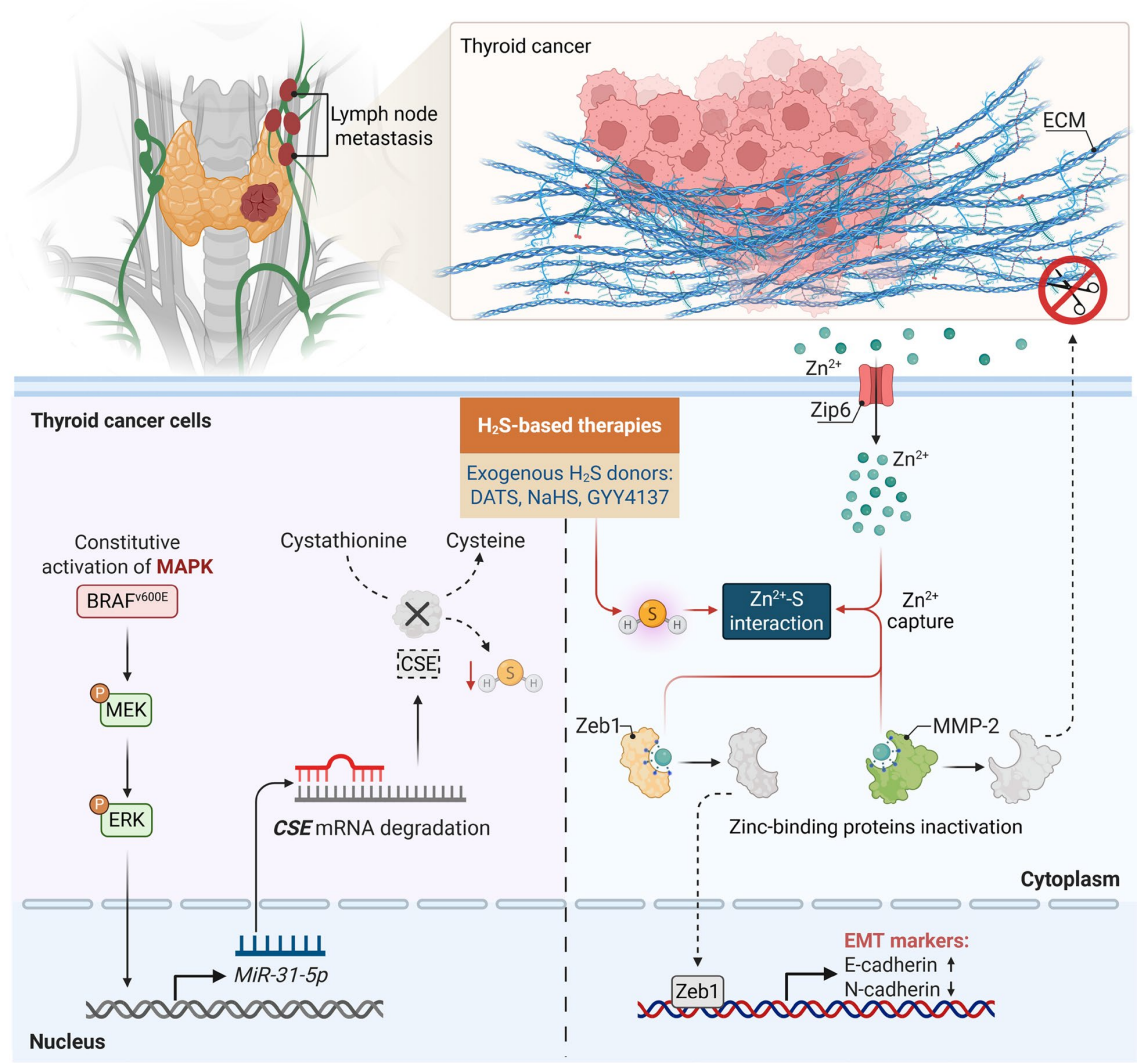

**Supplementary Information:**

The online version contains supplementary material available at 10.1186/s13046-026-03752-0.

## Introduction

Thyroid cancer incidence has risen globally over the past few decades, and differentiated thyroid cancer (DTC) accounts for the vast majority of these cases [1, 2]. While standard therapeutic interventions are highly curative for localized disease [3], up to 50% of patients present with or develop regional lymph node metastases [4], and distant metastases occur in roughly 10% of cases [5]. Once metastasis occurs, the clinical outcome worsens significantly, with the 5-year survival rate drastically dropping to approximately 48.3% [6]. This sharp decline highlights the urgent need to decipher thyroid cancer metastasis mechanisms to develop targeted therapeutic strategies for advanced disease.

The BRAF V600E mutation, occurring in about 60% of papillary thyroid carcinomas (PTCs) [1], constitutively activates the mitogen-activated protein kinase (MAPK) signaling pathway [7]. While this alteration is closely associated with clinically aggressive features, including lymph node metastasis, tumor dedifferentiation, and radioiodine refractoriness [8, 9], the intrinsic mechanisms through which it drives thyroid cancer progression remain incompletely understood. Accumulating evidence supports that coexisting BRAF V600E and *TERTp* mutations synergistically drive the most aggressive phenotypes, whereas each mutation alone has a modest effect [10]. Nevertheless, *TERTp* mutations are relatively infrequent, occurring in less than 10% of PTC cases [11]. This clinical phenomenon potentially suggests that the role of BRAF V600E in promoting disease progression has been somewhat overlooked, highlighting a critical need for further investigation.

Hydrogen sulfide (H_2_S) is a key gasotransmitter with pleiotropic physiological functions [12]. Initially, H_2_S has gained recognition as a critical regulator in cardiovascular pathophysiology, influencing arrhythmias, contractility, ischemia and vascular function *via* its interplay with nitric oxide signaling, protein S-sulfhydration, and redox homeostasis maintenance [13]. Beyond its cardiovascular functions, H_2_S plays a context-dependent role in cancer biology [14]. Dysregulation of H_2_S-producing enzymes in tumors disrupts physiological H_2_S homeostasis, resulting in either insufficient or excessive H_2_S levels, which can manifest both tumor-suppressive [15] and tumor-promoting effects [16]. To address this paradox specifically in thyroid cancer, our recent studies have delineated a tumor-suppressive role of H_2_S, showing its capacity to inhibit PTC cells growth, attenuate cancer stem cell phenotype, and restore thyroid-specific gene expression patterns in anaplastic thyroid cancer (ATC) cells [17, 18]. However, whether endogenous H_2_S homeostasis imbalance occurs in thyroid tumors and its potential role in disease progression remain unexplored.

It is well established that cystathionine γ-lyase (CSE, gene symbol *CTH*), cystathionine β-synthase (CBS), and 3-mercaptopyruvate sulfurtransferase (3-MST, gene symbol *MPST*) serve as the primary enzymes responsible for intracellular H₂S biosynthesis in mammalian systems [12]. To evaluate the endogenous H₂S status in thyroid cancer, we interrogated the TCGA database to profile the expression of these synthases. Notably, our analysis revealed that among these enzymes, only CSE was significantly downregulated in thyroid cancer tissues. Intriguingly, accumulating evidence reveals the upregulation of CSE in diverse malignancies, including prostate, colorectal, hepatic, and breast cancers, where it functionally promotes tumor initiation, progression, and therapeutic resistance [19]. This paradox prompted us to unravel the functional consequences and underlying mechanisms of CSE silencing in thyroid cancer progression.

In the present study, we elucidated that CSE/H_2_S system inactivation served as a pivotal molecular switch that couples BRAF V600E-driven MAPK hyperactivation to metastatic progression in thyroid cancer. We identified that BRAF V600E mutation induces *miR-31-5p*-mediated CSE/H_2_S deficiency, disrupting zinc homeostasis and activating Zeb1/MMP-2-driven metastasis. These mechanistic insights provide a foundation for developing H_2_S-based therapies to target metastatic progression in thyroid cancer.

## Materials and methods

### Chemicals, reagents and antibodies

Dabrafenib was obtained from MedChemExpress. Methyl thiazolyl tetrazolium (MTT) was purchased from Sangon (Shanghai, China). Hoechst 33,342, crystal violet and Lipo6000™ transfection reagent were purchased from Beyotime Biotechnology (Shanghai, China). The UltraSYBR mixture was obtained from CWBIO (Beijing, China). 3,3’-Diaminobenzidine (DAB) detection kit was purchased from MX Biotechnology (Fuzhou, China). Human H_2_S enzyme-linked immunosorbent assay (ELISA) kit (CJ-001193) was purchased from LanpaiBio (Shanghai, China). DATS, NaHS·xH_2_O, GYY4137, 7-Azido-4-methylcoumarin (AzMc), DL-Propargylglycine (PAG) and ZnCl_2_ were purchased from Sigma Aldrich (Saint Louis, Missouri, USA). The Matrigel Basement Membrane Matrix was purchased from Becton Dickinson (SanJose, California, USA). Transwell inserts (8.0 μm) were obtained from Merck Millipore (Massachusetts, USA). D-Luciferin, potassium salt was purchased from Yeasen Biotechnology (Shanghai, China). FluoZin3-AM was obtained from Thermo Fisher Scientific. The primary antibodies used in this research are listed in Table S3. All of the other chemicals were of the molecular biological grade and purchased from common sources.

### Cell culture

Normal thyroid follicular epithelial cell Nthy-ori-3.1 (RRID: CVCL_2659), ATC cell 8505C (RRID: CVCL_1054) were purchased from the European Collection of Cell Cultures (ECACC, Wiltshire, UK). Nthy-ori-3.1 cells were maintained in RPMI 1640 containing 10% fetal bovine serum (FBS). 8505C cells were maintained in MEM containing 10% FBS. PTC cell line B-CPAP (RRID: CVCL_0153), PDTC cell line KTC-1 (RRID: CVCL_6300), ATC cell lines BHT-101 (RRID: CVCL_1085), C643 (RRID: CVCL_5969) and Cal-62 (RRID: CVCL_1112) were obtained from National Collection of Authenticated Cell Cultures (Shanghai, China). B-CPAP cells were cultured with a complete medium containing 89% RPMI 1640, 10% FBS and 1% nonessential amino acids. KTC-1 cells were maintained in RPMI 1640 media containing 10% fetal bovine serum, 1% glutamine, 1% sodium salt pyruvic acid, 1% nonessential amino acids. BHT-101 cells were cultured in 80% DMEM and 20% FBS. C643 and Cal-62 cells were maintained in DMEM containing 10% FBS. PTC cell line TPC-1 (RRID: CVCL_6298) was purchased from Bena Culture Collection and maintained in 90% DMEM and 10% FBS. All cell culture media were supplemented with 100 U/ml penicillin, and 100 U/ml streptomycin. The cells were cultured in their respective media containing these antibiotics. All cell lines were incubated under a humid atmosphere of 5% (v/v) CO_2_ at 37 °C.

### Establishment of stable cell lines

Two shRNAs targeting the coding sequences (CDS) and 3’ untranslated regions (UTR) of *CSE* genes (NM_001902.6) were cloned into the *BamHI/EcoRI* sites of the miRZip lentivector (System Biosciences) according to the manufacturer’s recommendations. The target sequences for the shRNA constructs were as follows: for the human *CSE* shRNA targeting the CDS, the sequence was 5’-GCCCAGTTCCTGGAATCTAATC-3’; and for the human *CSE* shRNA targeting the 3’UTR, the sequence was 5’-GCACCTCATTATCTTTCATAACT-3’. The control scrambled (SCR) shRNA was engineered with sequence that mimics the structure of functional shRNAs but targeted no known human or mouse genes. The sequence of the scrambled shRNA was: 5’-TTCTCCGAACGTGTCACGT-3’.

A lentivirus producer cell line 293FT cells was transiently co-transfected with transfer (encoding shCSEs or scramble), envelope and packaging plasmids to generate lentiviral particles (30 µg of total DNA; 3:2:1 (wt/wt/wt) miRZip/psPAX2/ pMD2.G plasmid ratio). The virus was harvested and concentrated 72 h post transfection. Subsequently, TPC-1 cells were infected with the appropriate lentivirus in the presence of 10 µg/ml polybrene. Positive clones were then selected through puromycin (1 µg/ml) selection for two weeks. Limiting dilution cloning was performed to obtain individual cell clones, and CSE knockdowns were verified by western blot.

The full CDS of CSE (NM_001902.6) and its missense mutant CSE (E339A and D187A/T189A) were cloned into lentivector pcSLenti-EF1-EGFP-F2A-Puro-CMV-6xHis-WPRE. Lentivirus carrying the wildtype and mutant CSE transgenes were provided from OBiO Technology. CSE was stably overexpressed in KTC-1 cells via a lentiviral delivery approach. Positive clones were selected using puromycin (1 µg/ml) and limiting dilution cloning. CSE overexpression were verified by western blot analysis.

Firefly-derived luciferase bioluminescence was conducted to monitor thyroid cancer cells in vivo. A lentiviral vector (pASLenti-pA-LuC2-CMV-EF1-EGFP-P2A-Puro-WPRE) containing firefly luciferase (Luc), a puromycin-resistant gene, and enhanced green fluorescent protein (EGFP) was constructed for transfection of thyroid cancer cell lines and generation of stable clones (lentivirus was purchased from OBiO Technology). The luciferase activity was confirmed using ONE-Glo™ Luciferase Assay System (Promega) according to the manufacture’s instruction.

### Bioinformatics analysis

#### Overrepresented pathways and functional enrichment analysis on differentially expressed genes (DEGs)

Transcriptomic profiles and corresponding clinical data were retrieved from TCGA-THCA dataset. To capture robust molecular signatures driven by *CSE* expression, samples were ranked by *CSE* mRNA levels, and the top 20% were defined as the *CSE*^high^ cohort, while the bottom 20% constituted the *CSE*^low^ cohort. Differential expression analysis was performed using the *limma* R package. Genes satisfying the criteria of |log_2_ Fold Change| > 1.5 and an adjusted *P*-value < 0.05 were identified as DEGs. Functional annotation, including Gene Ontology (GO) and Kyoto Encyclopedia of Genes and Genomes (KEGG) analyses, was conducted using the *clusterProfiler* package (v4.4.3). For Gene Set Enrichment Analysis (GSEA), the complete gene list was ranked based on log_2_ Fold Change values. We utilized the MSigDB Hallmark gene sets (v2025.1, H category) *via* the *msigdbr* package to identify coordinated biological states. Statistical significance was determined using a False Discovery Rate (FDR/q-value) < 0.05.

#### Expression profiling of H_2_S-producing enzymes in thyroid cancer

The expression patterns of three key H_2_S-producing enzymes in thyroid cancers were initially analyzed using the Gene Expression Profiling Interactive Analysis (GEPIA) database (http://gepia.cancer-pku.cn/) [20]. To expand our analysis, we obtained gene expression data and corresponding clinical information from TCGA through both the UCSC Xena platform (https://xenabrowser.net/datapages/) and cBioPortal platforms (http://www.cbioportal.org/) [21]. For independent validation, we analyzed six Gene Expression Omnibus (GEO) datasets (GSE33630, GSE29265, GSE76039, GSE65144, GSE82208 and GSE53157) generated using the Affymetrix Human Genome U133 Plus 2.0 Array platform (GPL570) (http://www.ncbi.nlm.nih.gov/geo/) [22]. The raw CEL files underwent background adjustment and normalization using robust multichip average (RMA) method [23], with the RMA Express program accessible at the website http://rmaexpress.bmbolstad.com. Batch effects were corrected by ComBat method. All probes were aligned to the most current Affymetrix NetAffx annotation file. For gene sets targeted by multiple probes, their expression levels were calculated by averaging probe values.

#### Integrated spatial-single cell analysis

The spatial transcriptomic (ST) data and matched single-cell RNA sequencing (scRNA-seq) data of thyroid cancer were obtained from the GEO under accession number GSM7980866 [24]. Initial processing was performed using the *Seurat* (v5.0) R package. For ST data, spots were filtered to retain those with > 200 detected features and < 25% mitochondrial content. For scRNA-seq data, cells were excluded if they possessed < 200 or > 6000 features, > 20% mitochondrial transcripts, or > 1% hemoglobin content. Both datasets were normalized using the *SCTransform* workflow. Following normalization, we performed Principal Component Analysis (PCA) on the top-ranked variable features to achieve linear dimensionality reduction. The resulting top 30 principal components were then embedded into a two-dimensional space *via* Uniform Manifold Approximation and Projection (UMAP) to visualize cell clusters. We categorized these clusters into twelve primary cell lineages. An anchor-based integration strategy was then utilized to project single-cell identities onto the spatial transcriptomic landscape. The annotated scRNA-seq dataset was utilized as a high-resolution reference to identify mutual nearest neighbors (MNNs) within the ST query *via* the *FindTransferAnchors* framework. These spatial-single cell anchors facilitated the probabilistic transfer of cell-type labels using the *TransferData* algorithm. Each spot was subsequently assigned a dominant cell type based on the maximum prediction probability score.

To quantify malignant progression, we implemented two distinct scoring systems. The EMT score was calculated using the *AddModuleScore* function based on the GSEA Hallmark Epithelial-Mesenchymal Transition gene set, serving as a proxy for the degree of mesenchymal transformation. Concurrently, a *CSE* signature was developed by integrating the top 50 genes demonstrating the strongest positive correlation with *CSE* expression within the TCGA-THCA cohort. To mitigate the inherently predictive limitations of standard unsupervised trajectory inference, supervised trajectory inference was conducted using *Monocle3*, with the EMT score guiding the dimensionality reduction process to capture the invasive continuum. Crucially, the trajectory was computationally anchored by defining the cells within the normal region as the root state. The dynamic erosion of the *CSE* signature along this biological axis was modeled using Loess regression, with the strength and significance of the association quantified by Pearson correlation.

To resolve regional molecular heterogeneities, four distinct architectural domains, including adjacent normal, capsule stroma, tumor core, and leading edge were manually defined on the Visium sections by a senior pathologist. Spatial coordinates for each spot within the annotated malignant domains were then computationally extracted and partitioned into their respective anatomical compartments. The normalized expression values of the *CSE* transcript and the calculated EMT scores were aggregated across all spots localized strictly within these defined coordinates. Then we conducted a targeted comparative analysis focusing on the tumor core and the leading edge to delineate the raw *CSE* expression and EMT score between distinct tumor compartments, using the Wilcoxon rank-sum test.

### Clinical sample collection

Formalin-fixed paraffin-embedded (FFPE) and fresh surgical human PTC samples were gathered from patients who underwent thyroidectomy at Jiangyuan Hospital, affiliated with Jiangsu Institute of Nuclear Medicine, between January 2021 to December 2023. Those tissues with diameter > 2 cm were separated into tumor (T) and adjacent non-tumoral sections (NT) by the pathologists. The pathological types of all samples were verified by two independent experienced pathologists. The BRAF mutation status of the PTC specimens were determined by the Clinical Genetics Laboratory of Jiangyuan Hospital. The detailed demographic and clinicopathological characteristics of all enrolled patients, along with the specific sample allocation for each experimental assay, are summarized in Patient List.

The study was conducted in accordance with the ethical standards set forth in the Helsinki Declaration of 1975, as amended in 2000. All procedures were approved by the Institutional Review Board (or Ethics Committee) of Jiangsu Institute of Nuclear Medicine (protocol number: YL202120. 1st, January 2021). Specifically, all enrolled patients were fully informed about the objectives and procedures of the study, and provided their consent for the publication of their anonymized examination results and clinical information.

### Reverse transcriptase PCR (RT-PCR) and quantitative real-time PCR (qPCR)

Total RNA was isolated from thyroid cancer cells and human PTC samples using TRIzol extraction reagent (Invitrogen), and cDNA synthesized *via* reverse transcription. After amplification, PCR products were resolved on 1.5% agarose gel and visualized with ethidium bromide dying. Representative images were captured using the GIS-2019 system (Tanon, Shanghai, China).

Quantitative real-time PCR (qPCR) was performed using UltraSYBR dye mixture (CWBio). The mRNA levels of target genes were normalized to the house-keeping gene using 2^−ΔΔCT^ (Livak) method. The primer sequences for RT-PCR and qPCR used in this study were listed in Table S4.

### Hematoxylin-eosin (H&E), immunohistochemistry (IHC) and Immunofluorescence (IF) staining

H&E and IHC assays were performed as described previously [25].

For the IF assay, surgical PTC samples were promptly collected and flash-frozen at -80 °C. Embedded in optimal cutting temperature compound (OCT), the frozen tissues were sectioned into 8-µm-thick slices using a cryostat (MNT, SLEE medical GmbH). Slides were fixed and permeabilized with pre-cooled acetone for 20 min at RT. After blocking with 1% bovine serum albumin (BSA) for 1 h, the slices were incubated with the specific primary antibodies overnight at 4 °C, followed by incubation with the corresponding fluorophore-conjugated secondary antibodies. Nuclei were counterstained with Hoechst33342, and the slides were visualized under a fluorescent microscope (OLYMPIS, X51).

### Western blot analysis

Western blotting was performed as previously reported [26].

### Cell proliferation and viability assay

Cell proliferation was assessed through monitoring the cell numbers over time. Briefly, 100 µL of cancer cell suspension (10^5^ cells/well) were planted in a 12-well plate. At 24-h intervals for 7 consecutive days, cells were harvested, stained with trypan blue, and the number of unstained living cells was determined using an automated cell counter (BodBoge, Shenzhen, China). Cell viability was assessed using the MTT assay, following our standard protocols [27].

### Plasmids construction and transient transfection

The full-length CDS of both wildtype and missense mutant (c.1799T > A, p.Val600Glu) B-Raf proto-oncogene, serine/threonine kinase (BRAF) gene (NM_004333.6) were cloned into pCMV-N-Flag vectors. These constructs were transfected into thyroid cancer cells using Lipo6000 transfection reagent, according to the manufacturer’s recommendations. After a 24 h incubation, cells were harvested for protein extraction or total RNA isolation.

### Measurement of H_2_S production

#### Intracellular H_2_S measurement *via* fluorescence microscopy

Thyroid cancer cells were pre-treated with different dosages of H_2_S donors for 2–4 h, followed by incubation with 10 µM of AzMc fluorescent probe for 30 min. Intracellular H_2_S levels were then visualized and quantified using a fluorescence microscope (OLYMPUS, IX-81).

#### Extracellular H_2_S quantification *via* ELISA

To measure H_2_S levels in cell supernatants, a H_2_S ELISA Kit was employed as previously described [17].

#### H_2_S release detection *via* colorimetric assay

H_2_S release was detected using a colorimetric assay as previously described [18].

### Cell migration and invasion assay

The migratory and invasive abilities of thyroid cancer cells were evaluated through wound healing assays (for migration) and matrigel-coated transwell assay (for invasion), following previously described methods [27].

### Measurement of intracellular zinc levels

Cytosolic Zn^2+^ concentrations were measured using a fluorescent Zn^2+^-selective indicator FluoZin3-AM through confocal microscopy and flow cytometry. For microscopic analysis, thyroid cancer cells were seeded into glass-bottom culture dishes and cultured overnight to 70–80% confluence. Then, cells were pre-loaded with 10 µM of FluoZin3-AM in serum-free medium at 37 °C for 30 min, followed by treatment with different dosages of H_2_S donors. Cytosolic Zn^2+^ signal was monitored at indicated time points under a confocal fluorescence microscopy (OLYMPUS, IX-81). For flow cytometric analysis, cells were harvested and loaded with 10 µM of FluoZin3-AM at 37 °C for 30 min. Then the cells were washed with PBS and analyzed by a flow cytometer (FACS Calibur, Becton Dickinson). Fluorescence intensity was normalized to untreated controls and quantified using ImageJ 1.38 (microscopy) or FlowJo 10.0.7 (flow cytometry) software.

### MMP-2 activity analysis by gelatin zymography

MMP-2 enzymatic activity was measured using gelatin zymography. Briefly, thyroid cancer cells were cultured in 100-mm dishes with complete media until reaching 70–80% confluency. After washing twice with serum-free media, cells were maintained in serum-free conditions for 24 h to collect conditioned media. The media were centrifugated at 8,000 *g* for 5 min to remove cellular debris, and protein concentration was determined by BCA assay. Equal amounts of protein (50 µg) were mixed with 5 × non-reducing sample buffer (4% SDS, 20% glycerol, 0.01% bromophenol blue in 125 mM Tris-HCl, pH 6.8) and electrophoresed through 10% polyacrylamide gel containing 0.1% gelatin (prepared with 1% gelatin, 30% acrylamide/bis solution, 375 mM Tris-HCl pH 8.8, 0.1% SDS, 0.05% APS, and 0.05% TEMED). Following electrophoresis gels were washed by washing buffer (2.5% Triton X-100, 50 mM Tris-HCl, 5 mM CaCl_2_, 1 µM ZnCl_2_ and 0.1% NaN_3_, pH 7.5) for 1 h to remove SDS, then rinsed in incubation buffer (1% Triton X-100, 50 mM Tris-HCl, 5 mM CaCl_2_, 1 µM ZnCl_2_ and 0.1% NaN_3_, pH 7.5) for at 37 °C for 24 h with gentle agitation. Gelatinolytic activity was visualized by staining with 0.5% Coomassie Brilliant Blue R-250 for 30 min followed by decolorizing in 30% methanol/10% acetic acid until clear proteolytic bands appeared against the blue-stained gelatin background. MMP-2 activity was quantified by densitometric analysis of the cleared zones using ImageJ software.

### Prediction of Protein Structures and Molecular Docking

The three-dimensional structures of wildtype CSE and its mutants (E339A and D187A/T189A) were predicted using AlphaFold3 with high confidence scores [28]. The structure of the substrate, L-Cystathionine, was prepared and energy-minimized using the Molecular Operating Environment (MOE) software (v2022.02). Molecular docking was performed to simulate the interaction between L-Cystathionine and the CSE variants. The binding site was defined based on the canonical catalytic pocket of the wildtype enzyme, centered on the PLP-binding residue Lys212. Interaction analyses were visualized using the Ligand Interactions tool in MOE to identify key hydrogen bonds, salt bridges, and hydrophobic contacts.

### Animal studies

#### Animal husbandry

All animal procedures were performed in strict compliance with the National Institutes of Health Guide for the Care and Use of Laboratory Animals, and were approved by the Institutional Review Board of Jiangsu Institute of Nuclear Medicine. Female NCG mice (NOD/ShiLtJGpt-*Prkdc*^*em26Cd52*^*Il2rg*^*em26Cd22*^/Gpt, RRID: IMSR_GPT: T001475), aged 4-6-weeks, were purchased from GemPharmatech and housed in specific pathogen-free (SPF) facilities with ad libitum access to food and water at Jiangsu Institute of Nuclear Medicine.

#### IVIS imaging

Mice were randomized into treatment groups (Scramble, shCSE-CDS or shCSE-UTR; Mock or CSE-OE). Luciferase-expressing thyroid cancer cells (7.5 × 10^5^ cells in 0.25 ml of PBS) were intravenously injected *via* tail vein. Starting on day 7 post-injection, tumor progression was monitored and quantified weekly for a total of four weeks by IVIS Spectrum imaging (PerkinElmer) after intraperitoneally luciferin injection (3 mg/mouse). Mice were anesthetized with 2.5% isoflurane during imaging. The pseudocolor images representing the spatial distribution of detected photon counts emerging from the active luciferase within each animal were collected. Signal intensity was quantified using the IVIS Living Image software (Living Image 4.5.5). For ex vivo validation, organs were harvested post-CO_2_ euthanasia, imaged again with the IVIS system, and fixed in formalin for histological analysis.

#### In Vivo nuclear imaging of thyroid cancer metastasis

To investigate thyroid cancer lung metastases, we employed a multimodal nuclear imaging approach using radioiodine (^131^I), ^18^F-labelled fluorodeoxyglucose (^18^F-FDG) and ^68^Ga-labelled Arg-Gly-Asp (^68^Ga-NOTA-RGD) tracers. The radiolabeled compounds (^18^F-FDG and ^68^Ga-NOTA-RGD) were prepared as previously described [29, 30], with radiochemical purity (> 99%) confirmed by RP-HPLC analysis using a Waters Breeze system equipped with a Supersil C18 column (5 μm, 250 × 4.6 mm, Elite), a Radiomatic 610TR flow scintillation analyzer (PerkinElmer), and a Waters 2487 dual λ absorbance detector. The mobile phase was a trifluoroacetic acid/acetonitrile gradient system (1 ml/min flow rate). PET imaging was performed at 60–20 min after intravenous administration of 3.7 MBq (100 µCi) of ^18^F-FDG or ^68^Ga-NOTA-RGD using a 9.4 T Bruker BioSpec PET/MR system (Bruker Biospin, Ettlingen, Germany). Notably, the preparation and care of mice for PET imaging with ^18^F-FDG were performed according to the recommendations proposed by Veloso et al. [31]. SPECT/CT imaging (Albira Si, Bruker Biospin, Ettlingen, Germany) included a 1-h dynamic scan post-^131^I intravenous injection (3.7 MBq) followed by 30-minute static scans at 24 and 48 h. All images were reconstructed using 3-dimensional ordered subset expectation maximum algorithm and analyzed with PMOD software (v.4.4).

### Statistical analysis

Comparisons between two groups were performed using two-sided Student’s *t*-test. For multiple comparisons, one-way ANOVA followed by Dunnett’s post hoc test were conducted. A two-way ANOVA was employed to analyze the effects of two independent categorical factors on a continuous dependent variable. For non-parametric comparison of median differences across three or more independent groups, the Kruskal-Wallis test was employed. All statistical analyses were performed using GraphPad Prism (v9.0.0) or SPSS (v20.0), with a significant threshold of *p* < 0.05. The number of replicates for each experiment was noted in the corresponding figure legends.

## Results

### CSE is aberrantly downregulated in thyroid cancer and correlates with aggressive clinicopathological features

**Fig. 1:**
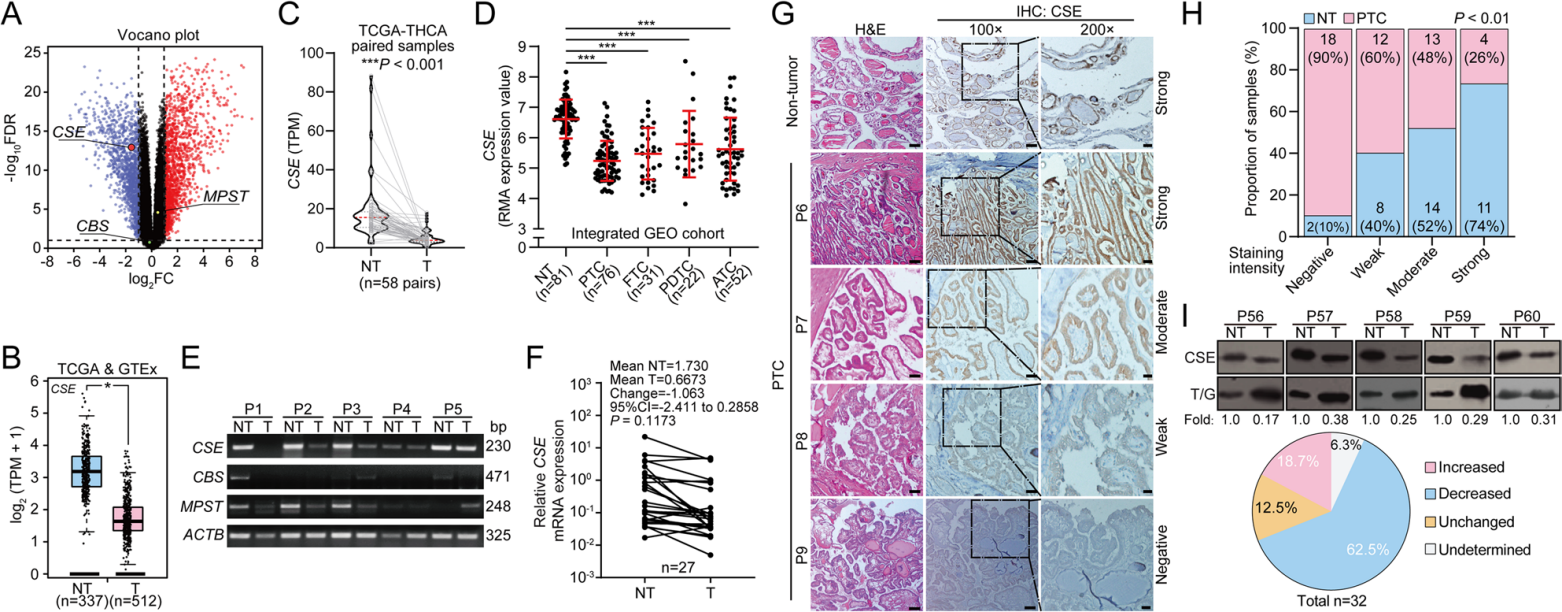
CSE is aberrantly downregulated in thyroid cancer and correlates with aggressive clinicopathological features. **A** Volcano plot of differentially expressed genes between 65 PTCs and their matched non-tumoral tissues obtained from TCGA database. The three H_2_S-producing enzymes were marked in the plot. **B***CSE* expression profiles in PTC (T) and non-tumoral (NT) tissues from TCGA and GTEx databases. *P < 0.05, Student’s *t*-test. **C** The mRNA expression (TPM) of *CSE* in 58 pairs of matched thyroid tumoral/non-tumoral samples from TCGA-THCA dataset. ***P < 0.001, two-tailed Student’s paired *t*-test. **D** RMA-normalized *CSE* expression across NT, PTC, FTC, PDTC and ATC samples in 6 integrated GEO datasets (GSE33630, GSE29265, GSE76039, GSE65144, GSE82208, and GSE53157). ***P < 0.001, Kruskal-Wallis test. **E** RT-PCR analysis of *CSE*, *CBS*, and *MPST* mRNA in 5 matched thyroid tumoral/non-tumoral pairs from Jiangyuan Hospital cohort. **F** QPCR analysis of *CSE* mRNA in 27 matched thyroid tumoral/non-tumoral pairs from Jiangyuan Hospital cohort. *P* = 0.1173, two-tailed Student’s paired *t*-test. **G** Representative images of HE/IHC staining of CSE in PTC specimens from Jiangyuan Hospital cohort. Scale bar, 100 μm. **H** Frequency distribution of CSE IHC staining intensity (*n* = 47; χ²= 11.9, *P* < 0.01). **I** Representative western blot images of CSE in 32 paired PTC (T) and adjacent non-tumoral (NT) tissues from Jiangyuan Hospital cohort. T/G: Tubulin or GAPDH. A pie chart was presented below to illustrate the changes in CSE protein levels

Building upon the tumor-suppressive effects of H₂S in thyroid cancer [17, 18], we sought to determine the expression profiles of endogenous H₂S-producing enzymes in PTC. Analysis of the TCGA-THCA dataset revealed that only *CSE* demonstrated significant differential expression (|log_2_FC| > 1, FDR < 0.05) between tumoral and normal tissues (Fig. 1A). Validation across combined TCGA and GTEx datasets confirmed widespread *CSE* downregulation in PTC (*n* = 512) compared to normal thyroid tissues (*n* = 337) (Fig. 1B), whereas *CBS* and *MPST* showed no significant differences (Fig. S1A, B). When compared with matched normal tissues, PTC tumors showed markedly reduced *CSE* expression in both the TCGA-THCA and GEO cohorts (Fig. 1C and Fig. S1C). Expanding beyond PTC, we analyzed a comprehensive GEO dataset encompassing all major histotypes. As shown in Fig. 1D, *CSE* expression was consistently downregulated across all histotypes of thyroid cancer, including PTC, follicular thyroid cancer (FTC), poorly differentiated thyroid cancer (PDTC), and ATC. Notably, *CSE* expression in normal thyroid tissue ranks among the highest in the human body, underscoring its critical role in maintaining thyroid homeostasis (Fig. S1D).

We next validated these findings in our local patient cohort. RT-PCR demonstrated pronounced silencing of *CSE* in thyroid cancer tissues compared to paired adjacent normal tissues. Among these five thyroid cancer samples, *CBS* was undetectable in three (3/5), decreased in one (1/5), and slightly increased in one (1/5) (Fig. 1E). While *MPST* downregulation was also observed in a subset of our samples (contrasting with TCGA data), *CSE* showed the most consistent downregulation based on both public datasets and our preliminary screening. Consequently, we prioritized CSE for functional characterization. QPCR analysis in our cohort revealed a downward trend of *CSE* in tumors relative to their paired normal tissues, although the difference did not reach statistical significance (Fig. 1F). Receiver operating characteristic (ROC) curve analysis of *CSE* mRNA levels suggested that *CSE* levels possessed moderate diagnostic potential for distinguishing malignant from benign nodules (AUC = 0.667; Fig. S1E), though validation in larger cohorts is required.

At the protein level, IHC staining of 82 PTC tissues confirmed a significant downregulation of CSE in tumors compared to adjacent normal tissues (Fig. 1G), with the majority of tumors exhibiting negative-to-weak staining (Fig. 1H). This pattern was consistent across multiple detection platforms, as corroborated by tissue microarray data from the Human Protein Atlas (Fig. S1F) and immunofluorescence (IF) assays (Fig. S1G). Furthermore, western blot analysis of paired surgical specimens verified reduced CSE protein abundance in 62.5% (20/32) of cases (Fig. 1I). Clinically, interrogation of the TCGA-THCA dataset revealed that low *CSE* expression is significantly associated with aggressive PTC clinicopathological features, including lymph node metastasis and the aggressive tall cell subtype (Table S1).

Given that the loss of thyroid-specific characteristics is a hallmark of aggressiveness, we further investigated the relationship between *CSE* and tumor differentiation. TCGA-THCA cohort analysis demonstrated a substantial positive correlation between *CSE* expression and the thyroid differentiation score (TDS) (Pearson *r* = 0.60, Fig. S2A). Functional manipulation of CSE in vitro confirmed its critical role in maintaining thyroid cancer differentiation and radioiodine uptake capacity (Fig. S2B, C). These collective findings highlight that CSE downregulation is a critical clinical event in PTC progression, which directly prompted us to investigate the primary upstream regulator responsible for this deficiency.

### BRAF V600E mutation drives downregulation of CSE

**Fig. 2:**
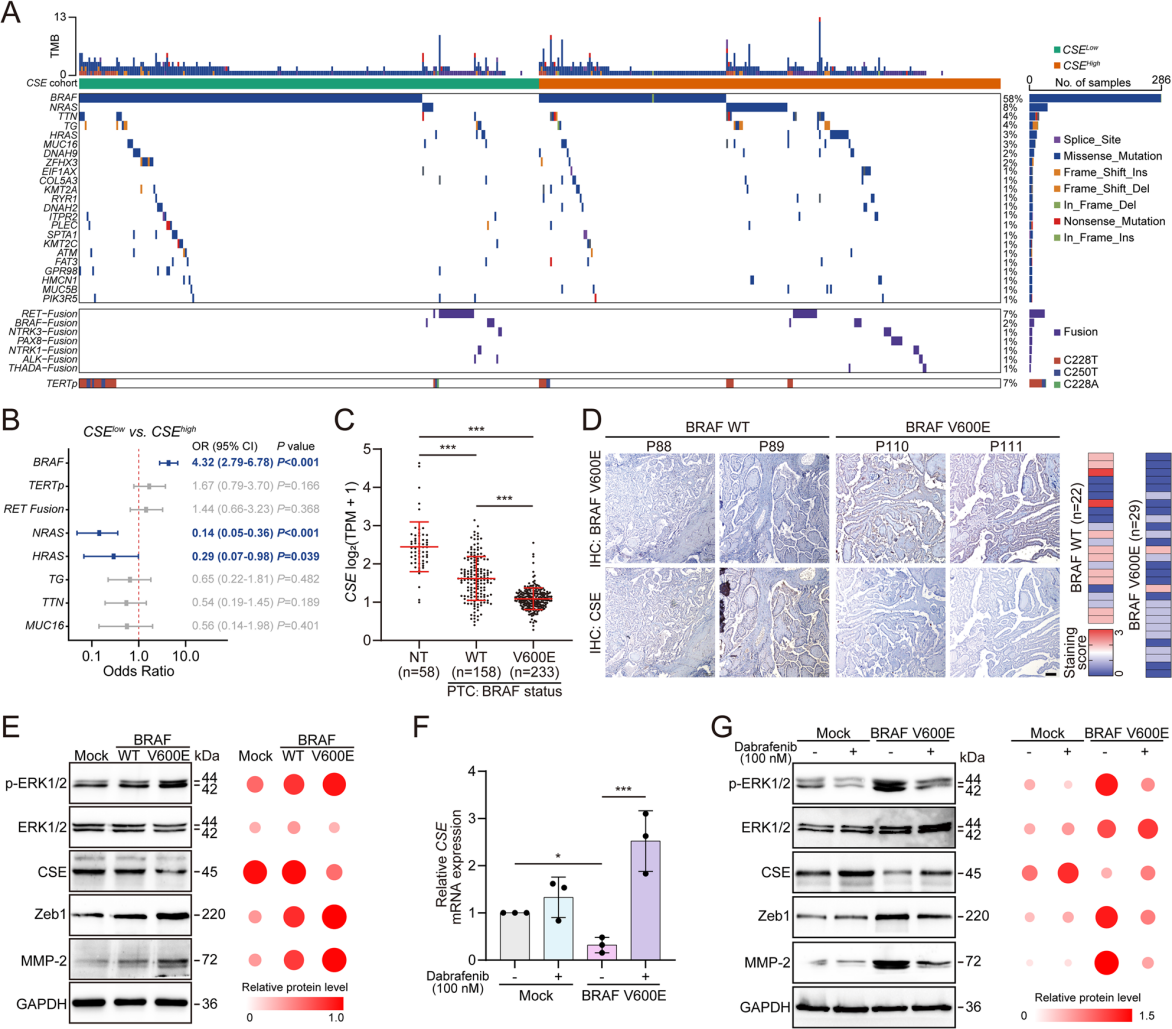
BRAF V600E mutation drives downregulation of CSE. **A** Comprehensive mutational landscape of the TCGA-THCA cohort stratified by *CSE* expression status. The streamlined oncoprint waterfall plot displays the most prevalent genomic alterations, restricted to exonic point mutations with > 5 mutational events, alongside kinase gene fusions and *TERTp* mutations. The top bar plot indicates the tumor mutational burden (TMB), and the right panel specifies the alteration frequency for each gene. **B** Forest plot illustrating the differential variant analysis between the *CSE*^*low*^ and *CSE*^*high*^ cohorts. Major genomic events with an overall incidence of > 3% were evaluated. Data are presented as odds ratio (OR) with 95% Confidence Intervals (CI) and FDR-adjusted *P* values. **C** Comparison of *CSE* mRNA expression levels across non-tumoral thyroid, BRAF wildtype (WT) and BRAF V600E-mutated PTCs in TCGA-THCA dataset. ***P < 0.001, Kruskal-Wallis test. **D** Representative IHC staining images of CSE and BRAF V600E in human BRAF WT (*n* = 22) and BRAF V600E-mutated (*n* = 29) PTC surgical specimens from our patient cohort. Scale bar: 100 μm. The right panel displays a heatmap of CSE staining intensity scores (1 = weak, 2 = moderate, 3 = strong) for each individual patient. **E** Western blot analysis of p-ERK, ERK, CSE, Zeb1 and MMP-2 in TPC-1 cells with BRAF WT or BRAF V600E overexpression. Right panel: Corresponding densitometric quantification visualized as a bubble plot. Both the size and color intensity of each bubble corresponded to the relative fold change of protein expression normalized to GAPDH. **F** Dabrafenib reverses BRAF V600E-mediated suppression of *CSE* mRNA in TPC-1 cells. Cells were transfected with mock or BRAF V600E plasmids and then treated with 100 nM of dabrafenib for 24 h, followed by qPCR analysis of *CSE* mRNA levels. *P < 0.05, one-way ANOVA. **G** Western blot analysis of p-ERK, ERK, CSE, Zeb1 and MMP-2. TPC-1 cells were treated as described in panel (**E**). In the corresponding densitometric bubble plot, both the size and color intensity of each bubble represent the relative fold-change of protein expression normalized to GAPDH (right panel)

To systematically identify the potential genetic contributors underlying CSE downregulation, we first extracted the comprehensive mutational landscapes of both the *CSE*^*high*^ and *CSE*^*low*^ cohorts. The genomic profiles are visualized in a stratified oncoprint waterfall plot, highlighting the most prevalent alterations (Fig. 2A). Subsequently, we performed a differential variant analysis using the *maftools* package to evaluate all major genomic events with an overall incidence of ≥ 3% across the cohort. Under stringent statistical thresholds (FDR-adjusted *P* < 0.05 and Odds Ratio > 2.0), BRAF V600E emerged as the sole genomic event significantly enriched in the *CSE*^*low*^ cohort (Fig. 2B). This critical finding indicates that the *CSE*^*low*^ phenotype represents a specific molecular state primarily linked to BRAF hyperactivation. To validate this hypothesis, we first assessed the basal protein levels of CSE across a panel of thyroid cell lines. As shown in Fig. S3A, CSE expression closely correlated with BRAF status: high levels were maintained in BRAF-wildtype cell lines (TPC-1, C643 and Cal-62), whereas significant suppression was observed in BRAF V600E-mutant lines (KTC-1, BCPAP, 8505C and BHT101). Stratification of TCGA samples showed that while *CSE* was reduced in BRAF-wildtype tumors compared to normal tissues, it underwent the most profound suppression in BRAF V600E-mutant PTCs (Fig. 2C). This pattern was recapitulated in inducible BRAF V600E transgenic mouse models (GSE58689 and GSE84650; Fig. S3B, C), which was further supported by IHC staining in our patient cohort (Fig. 2D). Specifically, 93.1% (27/29) of BRAF V600E-mutant PTCs displayed negative-to-weak CSE staining, compared to 50% (11/22) in the wildtype group (Fig. 2D). Furthermore, we extended this analysis to ATC. *CSE* mRNA levels (TCGA-MSKCC [32] and TCGA-GATCI [33] datasets) exhibited a downward trend in BRAF V600E-mutated ATC compared to wild-type samples, although this did not reach statistical significance. At the protein level (HRA007805 dataset [34]), while CSE was profoundly downregulated in ATC overall, its expression remained comparable between the BRAF V600E and wild-type subgroups (Fig. S3D-F). This difference from our findings in PTC likely reflects both the limited sample sizes of available ATC cohorts and the distinct biological nature of these two tumor entities [35].

Mechanistically, ectopic expression of BRAF V600E in TPC-1 cells constitutively activated the MAPK pathway, evidenced by increased ERK1/2 phosphorylation, which further resulted in the suppression of CSE and the induction of Zeb1 and MMP-2 (Fig. 2E). Moreover, these alterations were strictly dependent on BRAF signaling, as they were significantly abrogated by the specific BRAF V600E inhibitor dabrafenib (Fig. 2F, G). These findings reinforce the notion that CSE expression is negatively regulated by the hyperactive BRAF/MAPK signaling pathway.

### Loss of CSE facilitates the metastatic progression of PTC

**Fig. 3:**
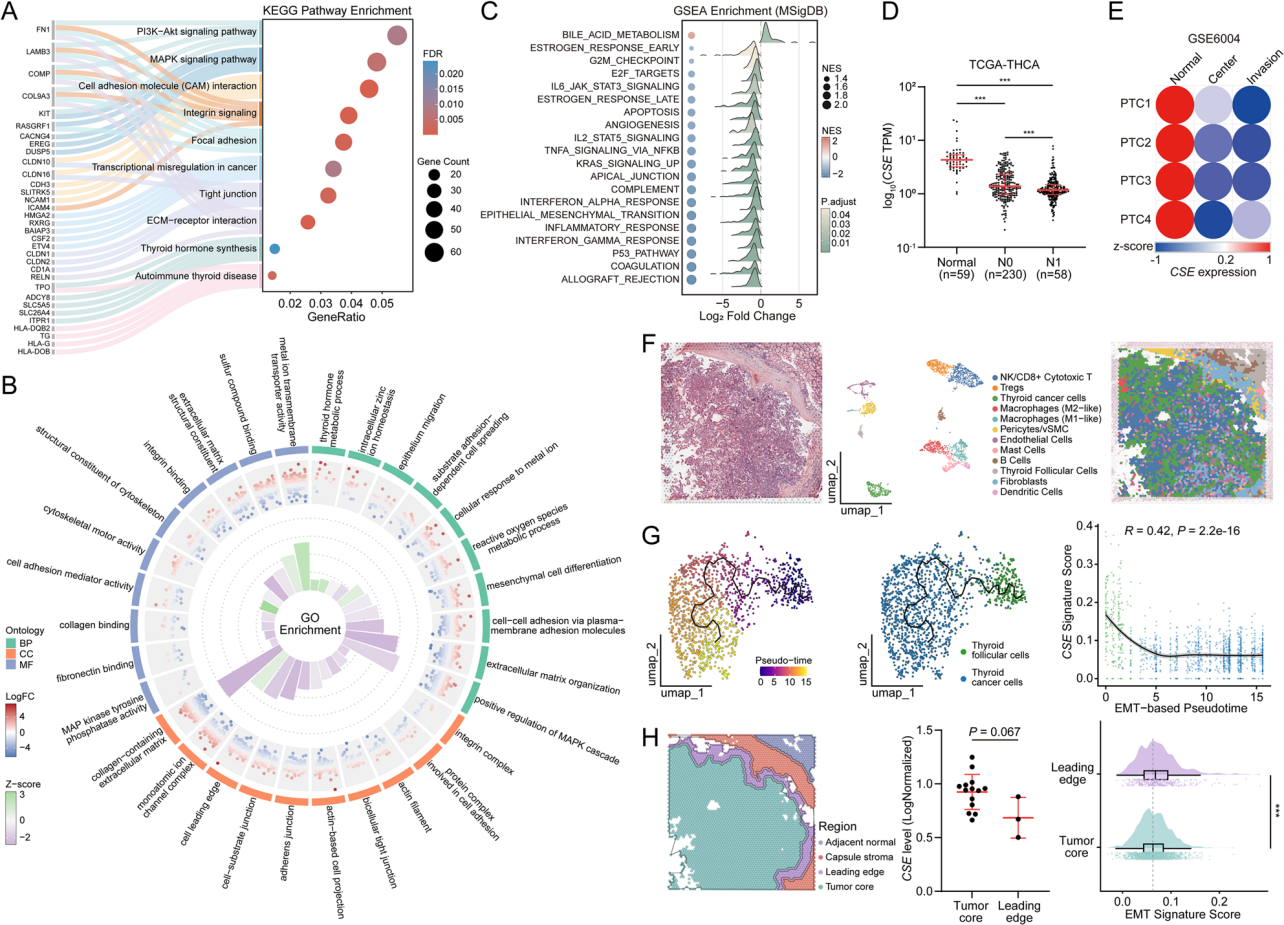
Loss of CSE facilitates the metastatic progression of PTC. **A** KEGG pathway enrichment analysis visualized as a Sankey-bubble diagram. The Sankey ribbons illustrated the linkage between the top differentially expressed genes and enriched pathways. Bubble color displayed the FDR. Bubble size represented the number of enriched genes. **B** Circular visualization of multi-dimensional GO enrichment results. The outer ring classified terms into biological processes (BP), cellular components (CC), and molecular functions (MF). The middle ring displayed individual enriched genes as a scatter plot, with color gradients reflecting their respective LogFC. The inner ring featured the z-score for each term, indicating the overall directional trend of the pathway (activation or suppression), where bar height represents the number of enriched genes. **C** Density ridge plot of GSEA results. The bubbles on the left indicated the normalized enrichment score (NES). The ridges illustrated the fold-change distribution of core enrichment genes within significant pathways. **D***CSE* mRNA expression across non-tumoral and PTC tissues with or without lymph node metastasis (TCGA-THCA). ***P < 0.001, Kruskal-Wallis test. **E** Heatmap analysis of *CSE* mRNA levels between the center and invasive front of PTC tissues (GSE6004 dataset). **F** Left panel: H&E-stained section of the thyroid carcinoma tissue used for Visium spatial transcriptomics (GSM7980866). Middle panel: UMAP visualization of the integrated scRNA-seq dataset, serving as the reference for cell type deconvolution. Right panel: spatial map of cell types. **G** Left panel: supervised pseudotime trajectory analysis of thyroid follicular cells based on EMT scores. Middle panel: spot annotations ranging from normal to tumor regions. Right panel: dynamic expression profile of the *CSE* signature along the EMT-driven pseudotime trajectory. **H** Left panel: manual segmentation of four ROIs on the tissue section. Middle and right panels: quantitative comparison of *CSE* raw counts and EMT scores between the tumor core and the leading edge within the defined ROIs. Representative spatial images demonstrating the distribution of these transcripts overlaid onto the corresponding ROIs are provided in Supplementary Fig. S4B. ***P < 0.001, Kruskal-Wallis test

To elucidate the CSE-associated molecular events in PTC progression, we stratified the TCGA-THCA cohort and compared the samples with the top 20% *CSE* expression (*CSE*^*high*^) to those with the bottom 20% (*CSE*^*low*^). Functional enrichment analysis (KEGG and GO) revealed that *CSE*^*high*^ phenotype was characterized by the enrichment of terms related to sulfur compound binding and zinc ion homeostasis, which aligned with the enzymatic function of CSE and pointed to its potential role in regulating intracellular zinc dynamics. Conversely, pathways associated with tumor aggressiveness, such as cell adhesion, cell-substrate junctions and extracellular matrix (ECM) organization were markedly inactive in the *CSE*^*high*^ group (Fig. 3A, B). Reinforcing these findings, Gene Set Enrichment Analysis (GSEA) demonstrated that aggressive oncogenic hallmarks, specifically angiogenesis and epithelial-mesenchymal transition (EMT), were significantly enriched in *CSE*^*Low*^ tumors (Fig. 3C). These data suggest that CSE may restrain thyroid cancer metastasis through the regulation of zinc metabolism and subsequent inhibition of EMT.

Clinically, *CSE* expression exhibited a stepwise downregulation that mirrored disease aggressiveness. In the TCGA-THCA cohort, *CSE* levels were significantly reduced in lymph node-negative (N0) tumors compared to normal tissues, with a further distinct suppression observed in lymph node-positive (N1) cases (Fig. 3D). This inverse association with local invasion was corroborated in micro-dissected samples (GSE6004), where *CSE* expression was significantly lower in the invasive fronts compared to the central core of primary tumors (Fig. 3E). Consistently, tumors with capsular invasion displayed markedly lower *CSE* levels than their encapsulated counterparts (Fig. S4A).

Subsequently, we performed a multivariate logistic regression analysis evaluating the risk of lymph node metastasis to statistically disentangle the specific contribution of *CSE*. Notably, even after stringently controlling for BRAF mutational status and classical clinicopathological parameters (including age, gender, and T-stage) [36], low *CSE* expression emerged as a highly potent and independent risk factor for lymph node metastasis (OR = 1.91, 95%CI: 1.28–2.85, *P* = 0.001; Table S2).

To characterize the spatial heterogeneity of thyroid carcinoma and further map this independent pro-metastatic factor, we integrated Visium ST with matched single-cell RNA sequencing (scRNA-seq) data (GSM7980866). Guided by histological H&E staining and canonical markers from the scRNA-seq reference (Fig. 3F), cellular composition was deconvolved into twelve distinct cell lineages. To capture the dynamic acquisition of metastatic potential and avoid computational predictions, we performed a supervised pseudotime inference on the subset of thyroid follicular and cancer cells strictly guided by the GSEA Hallmark EMT gene set. We intentionally defined the normal follicular cells as the root state to establish a stable epithelial baseline. Therefore, the resulting trajectory represents the continuous evolution of the EMT program rather than the chronological timeline of primary tumor formation. Along this EMT-based biological axis, a significant depletion of *CSE* signaling was observed (Fig. 3G). Beyond the overall negative correlation (*R* = -0.42), the pseudotime kinetics revealed that *CSE* loss acted as an early molecular trigger for the EMT process, coinciding with the transition from normal follicular cells to carcinoma cells. Because our data demonstrated that the downregulation of *CSE* is driven by the BRAF V600E mutation, the acquisition of this alteration during thyroid carcinogenesis logically explains the sharp decrease of *CSE* observed at this transition phase. This suppression reached a steady-state plateau as cells acquired a mesenchymal phenotype.

To validate this finding within the spatial context, we manually partitioned the tissue into four ROIs (Fig. 3H). Spatial quantification demonstrated that *CSE* transcript levels were significantly lower in the leading edge compared to the tumor core, while the EMT signature score exhibited a significant reciprocal increase at the leading edge. The inverse relationship between *CSE* and EMT was further visualized at the single-spot resolution (Fig. S4B). Collectively, these results suggest that the loss of *CSE* is a spatially-resolved hallmark of thyroid cancer progression.

### CSE expression negatively regulates the EMT and metastatic phenotypes in thyroid cancer cells

**Fig. 4:**
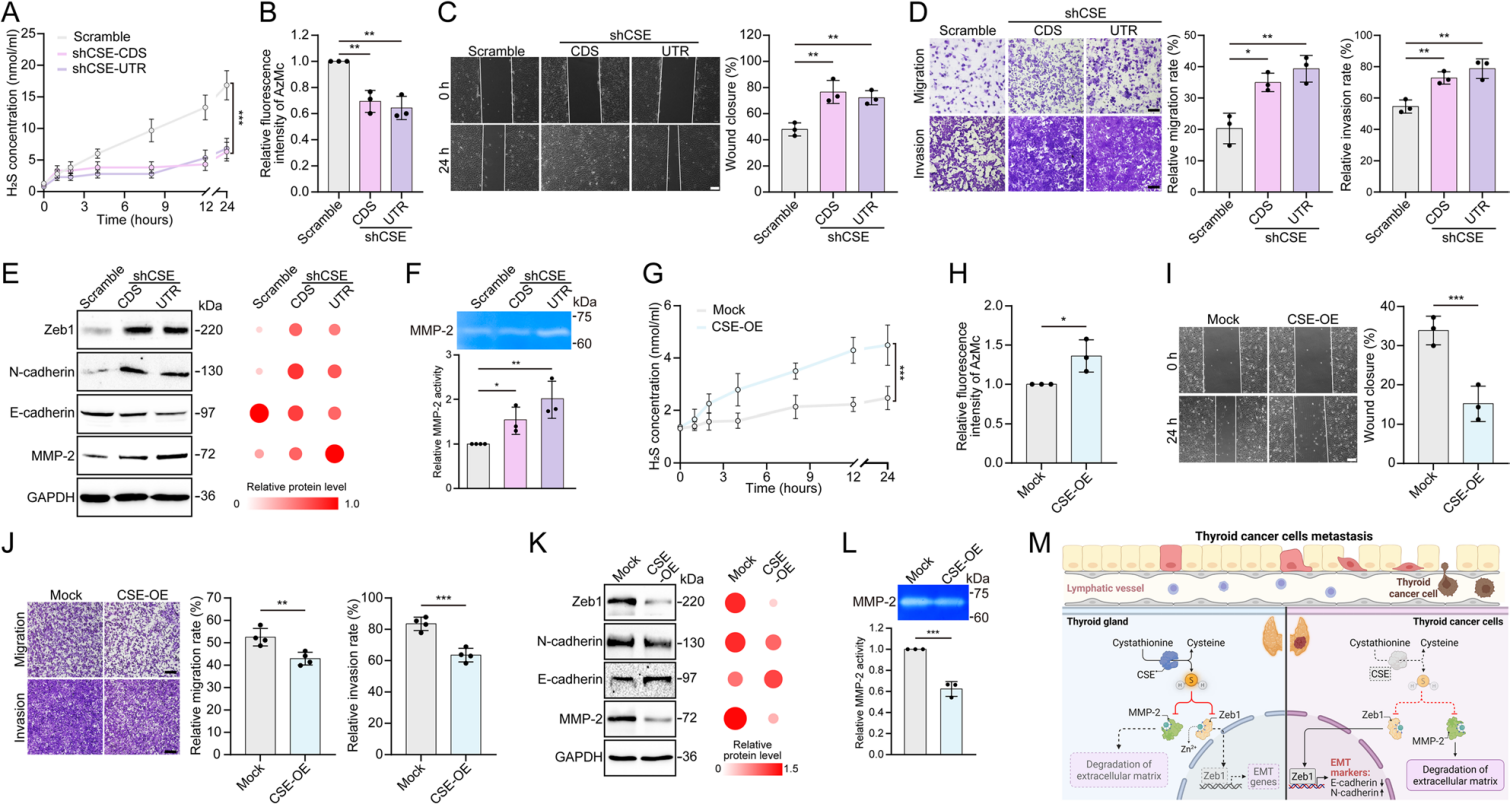
CSE expression negatively regulates the EMT and metastatic phenotypes in thyroid cancer cells. **A** Time-course analysis of intracellular H_2_S production in CSE-knockdown TPC-1 cells. ***P < 0.001, two-way ANOVA. **B** AzMc-based quantification of intracellular H_2_S in CSE-knockdown TPC-1 cells. **P < 0.01, one-way ANOVA. **C** CSE silencing promotes thyroid cancer cell migration in wound healing assays. Scale bar, 50 μm. Quantitative analysis of wound closure rate was shown in the right panel. **P < 0.01, one-way ANOVA. **D** Transwell migration/invasion assays with CSE-knockdown TPC-1 cells. Scale bar, 50 μm. Quantifications of migrated and invaded cells were shown on the right. *P < 0.05, **P < 0.01, one-way ANOVA. **E** Western blot analysis of Zeb1, N-cadherin, E-cadherin and MMP-2 in CSE-knockdown TPC-1 cells. Quantification of protein levels was visualized in the adjacent bubble plots, both the size and color intensity of each bubble represent the relative fold-change of protein expression normalized to GAPDH. **F** Gelatin zymography analysis of MMP-2 activity in the culture supernatants of CSE-knockdown TPC-1 cells. *P < 0.05, **P < 0.01, one-way ANOVA. **G** Time-course analysis of intracellular H_2_S production in CSE-overexpressed KTC-1 cells. ***P < 0.001, two-way ANOVA. **H** AzMc-based quantification of intracellular H_2_S in CSE-overexpressed KTC-1 cells. *P < 0.05, Student’s *t*-test. **I** CSE overexpression inhibits thyroid cancer cell migration in wound healing assays. Scale bar, 50 μm. ***P < 0.001, Student’s *t*-test. **J** Transwell migration/invasion assays with CSE-overexpressed KTC-1 cells. Scale bar, 50 μm. Quantitative analyses of cell migration and invasion were presented on the right. **P < 0.01, ***P < 0.001, Student’s *t*-test. **K** Western blot analysis of Zeb1, N-cadherin, E-cadherin and MMP-2 in CSE-overexpressed KTC-1 cells. Quantification of protein levels was visualized in the adjacent bubble plot, both bubble size and color intensity represent relative protein levels normalized to GAPDH. **L** Gelatin zymography analysis of MMP-2 activity in the culture supernatants of CSE-overexpressed KTC-1 cells. ***P < 0.001, Student’s *t*-test. **M** Diagram of how CSE silencing promoted thyroid cancer cell metastasis *via* H_2_S disruption, targeting Zeb1/MMP-2 activity

To define the biological function of CSE in thyroid cancer, we generated stable knockdown TPC-1 cell lines targeting the *CSE* CDS and 3’UTR, respectively. Functional validation confirmed that CSE silencing significantly reduced H_2_S production upon homocysteine stimulation (Fig. 4A, B) without affecting cell proliferation (Fig. S5A). However, CSE-knockdown cells exhibited a marked increase in migratory and invasive capabilities in both wound healing and transwell assays (Fig. 4C, D). In addition, CSE silencing triggered the downregulation of E-cadherin and upregulation of N-cadherin and markedly induced the expression of the zinc-finger transcription factor Zeb1 (Fig. 4E). Concomitantly, the protein levels of the zinc-dependent enzyme MMP-2 increased, and its proteolytic activity was significantly enhanced (Fig. 4F). Consistently, pharmacological blockade of CSE using the irreversible inhibitor DL-Propargylglycine (PAG) recapitulated the genetic knockdown phenotype. Specifically, PAG treatment inhibited intracellular H₂S levels and induced metastatic features in the absence of cytotoxicity (Fig. S5B-G). Conversely, stable overexpression of wildtype CSE in KTC-1 cells (KTC-1 CSE-OE) enhanced H₂S production (Fig. 4G, H and Fig. S5H), and significantly counteracted migration, invasion, the EMT progress and MMP-2 activity (Fig. 4I-L). These findings demonstrate that CSE-produced H_2_S acts as a critical negative regulator of EMT and metastatic potential in thyroid cancer cells (Fig. 4M).

### The anti-metastatic function strictly depends on CSE enzymatic activity and H₂S production

**Fig. 5:**
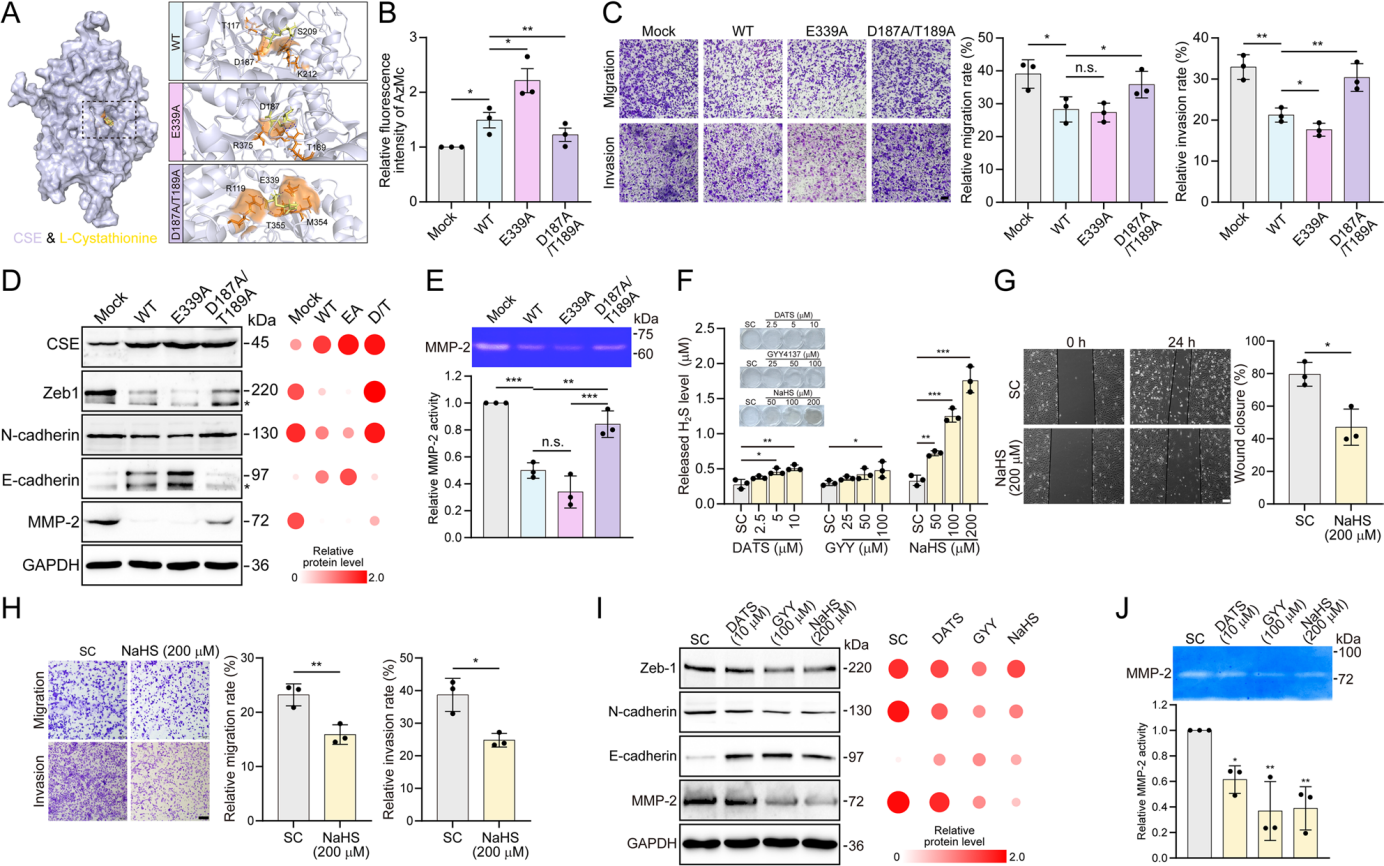
The anti-metastatic function strictly depends on CSE enzymatic activity and H₂S production. **A** Representative 3D docking poses of L-Cystathionine within the catalytic pocket of wildtype (WT) CSE and its mutants. Key amino acid residues interacting with the ligand were labeled, and the hydrogen bonds formed between them were depicted by blue dashed lines. **B** AzMc fluorescence assay quantified intracellular H_2_S production in KTC-1 cells expressing WT CSE or its mutants (c.1037 A > C/p.E339A, c.581 A > C/p.D187A and c.586 A > G/p.T189A double-position mutations). *P < 0.05, one-way ANOVA. **C** Transwell migration/invasion assays in KTC-1 cells overexpressing WT or mutant CSE. Scale bar, 50 μm. Quantification of migration or invasion cells was presented in the right panels. *P < 0.05, **P < 0.01, one-way ANOVA. **D** Western blot analysis of CSE, Zeb1, N-cadherin, E-cadherin and MMP-2 in KTC-1 cells transfected with WT or mutant *CSE* overexpression constructs. Right: Densitometric quantification plotted as bubbles (bubble size and color indicate GAPDH-normalized fold changes). **E** Gelatin zymography analysis of MMP-2 activity in the culture supernatants of KTC-1 cells expressing WT or mutant CSE. **P < 0.01, ***P < 0.001, n.s., no significance, one-way ANOVA. **F** H_2_S production in KTC-1 cells after treatment with H_2_S donors. Cells were treated with increasing dosages of DATS, GYY4137, or NaHS, and then H_2_S levels were quantified utilizing a colorimetric assay. *P < 0.05, **P < 0.01 and ***P < 0.001 *versus* control group, one-way ANOVA. SC, solvent control. Colorimetric assay image was inserted. **G** NaHS inhibits KTC-1 cell migration in wound healing assays. Scale bar, 50 μm. Quantification of relative wound closure rate was shown in the right panel. *P < 0.05, Student’s *t*-test. **H** NaHS inhibits the migration and invasion of KTC-1 cells in transwell assays. Scale bar, 50 μm. *P < 0.05, **P < 0.01, Student’s *t*-test. **I** H_2_S donors reduce EMT-related protein expression in KTC-1 cells. Cells were pre-treated with three distinct H_2_S donors for 24 h, and then the protein levels of Zeb1, N-cadherin, E-cadherin and MMP-2 were determined by western blot. Right: Densitometric quantification plotted as bubbles (bubble size and color indicate GAPDH-normalized fold changes). **J** H_2_S donors inhibit MMP-2 activity in KTC-1 cells. Cells were pre-treated with three distinct H_2_S donors for 24 h, and then the secretory MMP-2 activity was analyzed by gelatin zymography. *P < 0.05, **P < 0.01 *versus* solvent control group, one-way ANOVA

To further confirm that the metastatic phenotypes were governed by enzymatic activity, we then constructed specific CSE mutants, including the hyperactive E339A variant and the catalytically inactive D187A and T189A variants [37]. Structural modeling and docking analysis indicated that D187 acted as a critical gatekeeper residue in the wildtype CSE, anchoring the substrate *via* a hydrogen bond network. In the D187A/T189A mutant, the loss of this key interaction prevented the substrate from deeply penetrating the active pocket, resulting in a superficial and unstable binding mode. The removal of the glutamic acid side chain in E339A mutant relieved steric/electrostatic hindrance, allowing the recruitment of R375 to interact with the substrate (Fig. 5A). As shown in the 2D interaction map (Fig. S5I), R375 formed a new salt bridge with the substrate carboxyl group, which likely stabilized the substrate in an optimal orientation proximal to Lys212 (the PLP-binding residue) for efficient catalysis, while in the D187A/T189A mutant, the substrate is displaced from the catalytic center. As expected, the E339A mutant robustly produced H_2_S (Fig. 5B) and potently inhibited Zeb1 expression, MMP-2 activity, and cell migration (Fig. 5C-E). In contrast, the catalytically dead D187A/T189A mutant failed to generate H_2_S and lost the capacity to suppress EMT and metastasis.

Furthermore, we sought to determine whether pharmacological supplementation with exogenous H₂S donors (including DATS, GYY4137 and NaHS) could mimic the metastasis-suppressive effects of CSE. All three donors induced a dose-dependent release of H₂S (Fig. 5F). These donors exhibited minimal overall cytotoxicity, with significant viability reduction observed only at the highest doses of DATS and NaHS (Fig. S6A). As expected, we observed that all three H_2_S donors significantly suppressed migration and reversed EMT program caused by CSE silencing (Fig. 5G-J and Fig. S6B-E). These data confirm that H₂S is the indispensable bioactive effector responsible for CSE-mediated metastasis suppression.

### CSE-mediated suppression of thyroid cancer metastasis in mouse models

**Fig. 6:**
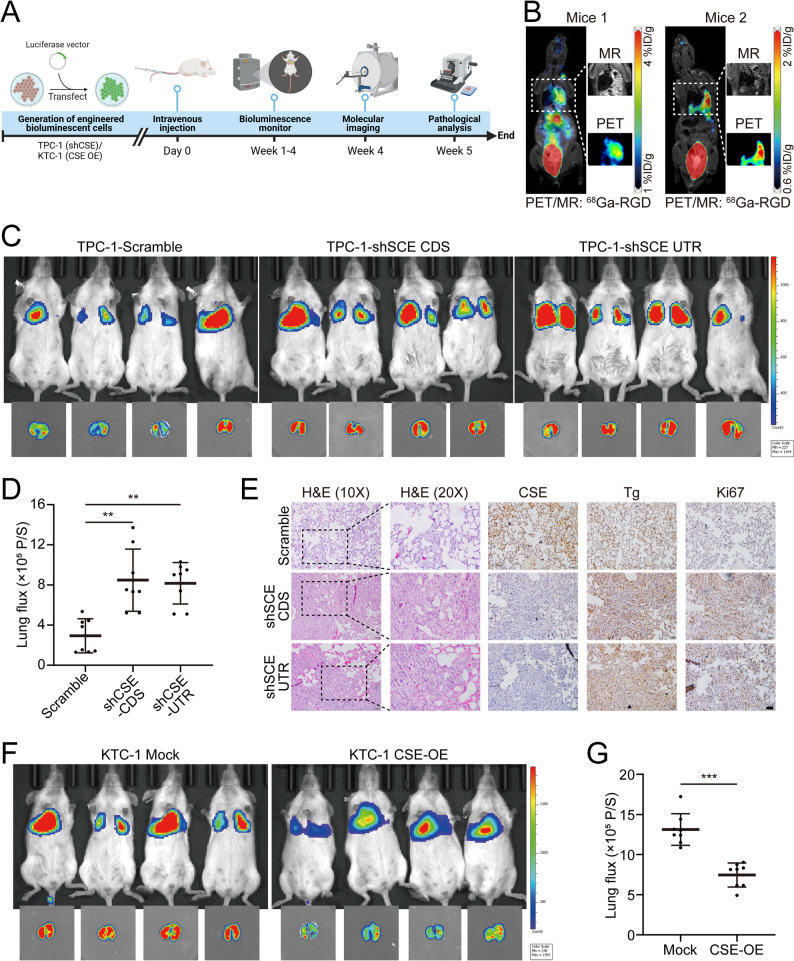
CSE-mediated suppression of thyroid cancer metastasis in mouse models. **A** Experimental scheme for evaluating the metastasis-inhibitory effect of CSE using an in vivo traceable metastatic mouse model. **B**^68^Ga-NOTA-RGD PET/MR imaging of thyroid cancer lung metastases in NCG mice. Mice were intravenously injected with 7.5 × 10^5^ of thyroid cancer cells to establish metastatic lesions, followed by 3.7 MBq of ^68^Ga-NOTA-RGD administration. Images were acquired at 20 min post-injection. Enlarged pulmonary MR and PET views were displayed in the right panels. **C** IVIS images of lung metastases in mice receiving intravenous injection of 7.5 × 10^5^ scramble or CSE knockdown TPC-1 cells. Pseudocolor images represented the bioluminescent signal intensity, reflecting spatial distribution of luciferase activity in vivo. Ex vivo images of lung were displayed below. **D** Quantification of average lung signal intensities in mice injected intravenously with scramble or CSE knockdown cells (*n* = 8 per group). P/S, photon radiance per second. **P < 0.01, one-way ANOVA. **E** Histopathological and immunohistochemical analyses of lung metastases after scramble or CSE knockdown cells injection. Representative H&E staining (shown at 10× and 20× magnifications) and IHC staining (CSE, Tg, and Ki67) of lung sections from mice euthanized 4 weeks post-injection were shown. Scale bar, 100 μm. **F** IVIS images of lung metastases in mice receiving intravenous injection of 7.5 × 10^5^ mock or CSE-overexpressed KTC-1 cells. Ex vivo images of lung were displayed below. **G** Quantification of average lung signal intensities in mice injected intravenously with mock or CSE-overexpressed KTC-1 cells (*n* = 8 per group). P/S, photon radiance per second. *P< 0.05, ***P < 0.001, Student’s *t*-test

To evaluate the impact of CSE on metastatic colonization in vivo, we established a lung metastasis model in immunodeficient NCG mice using distinct Luc2-eGFP-labeled thyroid cancer cells (Fig. 6A). Consistent with our in vitro experimental design, we utilized TPC-1 cells for CSE knockdown, and expanded our overexpression models to include KTC-1, Cal-62, and C643 cells. To achieve precise anatomical visualization of lung metastases using a clinically relevant diagnostic modality, we incorporated RGD (Arg-Gly-Asp)-based molecular imaging alongside longitudinal BLI. Given that RGD peptides specifically target integrins [38], we initially profiled the expression of these receptors to validate the feasibility of this imaging modality. Western blot analysis revealed a strong expression of the α2β1 integrin heterodimer in thyroid cancer cells compared to normal counterparts (Fig. S7A), providing a rationale for ^68^Ga-NOTA-RGD PET/MR imaging. Using the C643 model as a representative example, at 4 weeks post-injection, lung metastases were successfully visualized by RGD PET with high tumor-to-background contrast (Fig. 6B and Fig. S7B). Conversely, conventional ^131^I imaging was ineffective due to the loss of NIS expression, and ^18^F-FDG PET was compromised by high physiological uptake in the adjacent myocardium, which obscured metastatic lesions (Fig. S7C). Therefore, we established ^68^Ga-NOTA-RGD PET and bioluminescence imaging (BLI) as robust tools for tracking metastasis in our model.

Longitudinal BLI revealed that CSE silencing significantly exacerbated metastatic progression. By day 28, mice bearing CSE-knockdown TPC-1 cells exhibited a nearly two-fold increase in photon flux compared to the scramble control group (Fig. 6C, D). This exacerbated metastatic burden was further corroborated by ex vivo lung imaging and histological evaluation (Fig. 6E). H&E staining revealed that, rather than forming isolated or contained metastatic nodules, CSE-silenced tumor cells exhibited a highly aggressive pattern of diffuse infiltration and solid, sheet-like growth, leading to massive alveolar obliteration and severe parenchymal consolidation. IHC analysis confirmed the thyroid lineage of the metastatic lesions through strong Tg positive. Concurrently, the elevation of the Ki67 in CSE-silenced lung lesions provides compelling evidence of active metastatic colonization, as opposed to passive pulmonary entrapment or embolic sequestration resulting from the first-pass effect of the tail vein injection. Notably, the experimental procedure was well-tolerated, with no signs of severe systemic toxicity, weight loss, or overt morbidity observed (Fig. S7D, E). Conversely, CSE overexpression significantly attenuated lung colonization. CSE-overexpressing KTC-1 cells exhibited markedly reduced pulmonary bioluminescence signals (Fig. 6F, G). Furthermore, given the highly invasive and lethal nature of ATC, we sought to determine whether CSE could exert a universal metastasis-suppressive effect in the most aggressive histological subtype. Strikingly, our ATC models (Cal-62 and C643) also demonstrated noteworthy suppression of metastasis upon CSE overexpression (Fig. S7G). H&E staining further verified this suppression, showing preserved pulmonary architecture and reduced tumor infiltration (Fig. S7F, H). Collectively, these in vivo data provide compelling evidence that CSE functions as a potent suppressor of thyroid cancer metastasis.

### *MicroRNA-31-5p* mediates BRAF V600E-driven CSE suppression and promotes thyroid cancer cell metastasis

**Fig. 7:**
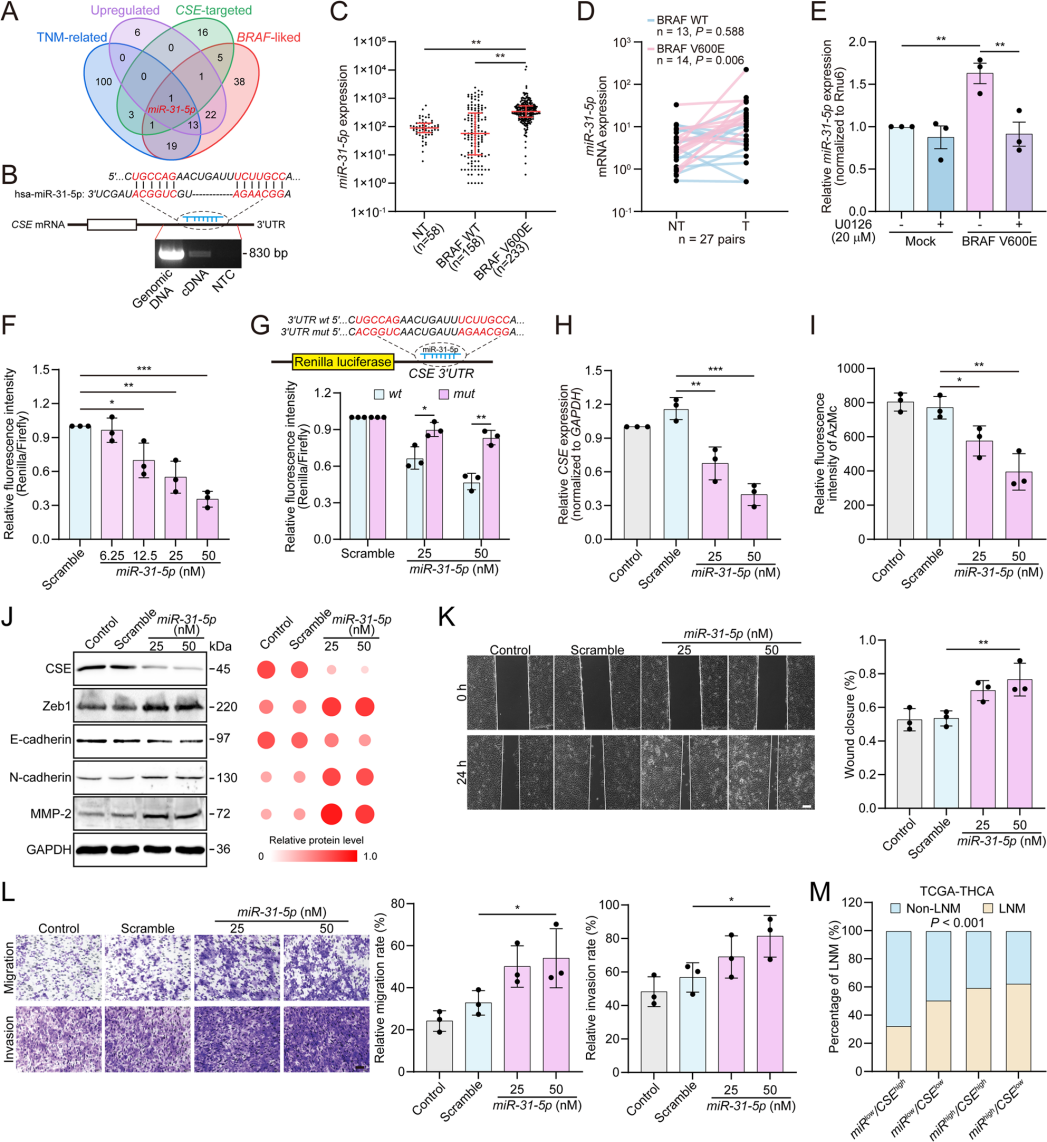
*MicroRNA-31-5p* mediates BRAF V600E-driven CSE suppression and promotes thyroid cancer cell metastasis. **A** Venn diagram of miRNAs linked to TNM staging, upregulated in tumors, targeting *CSE*, and regulated by BRAF V600E in thyroid cancer. **B** Targetscan-predicted *miR-31-5p* binding site at the 3’UTR of human *CSE* mRNA. The *CSE* 3’UTR region was verified by sequencing genomic DNA and cDNA samples obtained from TPC-1 cells. Schematic of predicted *miR-31-5p/CSE* 3’UTR interaction, with conserved seed sequence (red). **C***MiR-31-5p* expression levels in thyroid tissues stratified by BRAF V600E mutation status (TCGA-THCA). **P < 0.01, Kruskal-Wallis test. **D***MiR-31-5p* expression levels in PTC (T) and matched non-tumorous tissues (NT) from Jiangyuan Hospital cohort (*n* =27 pairs). Blue lines: BRAF-wildtype cases (*P* = 0.588, paired *t*-test). Red lines: BRAF V600E-mutated cases (*P* = 0.006, paired *t*-test). **E** BRAF V600E upregulates *miR-31-5p* through MEK/ERK signaling in TPC-1 cells. Cells were transfected with WT or BRAF V600E plasmids followed by 20 µM of U0126 treatment for 24 h, and then the mRNA level of *miR-31-5p* was analyzed by qPCR. **P < 0.01, one-way ANOVA. **F** Luciferase reporter assay validating *miR-31-5p* targeting of *CSE* 3’UTR. The 3’UTR of *CSE* was cloned downstream of *Renilla* luciferase gene in psiCHECK2 vector. TPC-1 cells were co-transfected with reporter construct and *miR-31-5p* mimics. Dual-luciferase activity was measured (Renilla/Firefly ratio). *P < 0.05, **P < 0.01, ***P < 0.001, one-way ANOVA. **G** Mutational analysis of *miR-31-5p* binding sites in *CSE* 3’UTR by dual-luciferase reporter assay. Site-directed mutagenesis was performed on predicted *miR-31-5p* binding sites in *CSE* 3’UTR. Wildtype (wt) or mutant (mut) 3’UTR sequence was cloned downstream of *Renilla* luciferase gene in psiCHECK2 vector. TPC-1 cells were co-transfected with wt or mut reporter plasmid and *miR-31-5p* mimics. Dual-luciferase activity was measured (Renilla/Firefly ratio). *P < 0.05, **P < 0.01, two-way ANOVA. **H** QPCR analysis of *CSE* levels in TPC-1 cells transfected with *miR-31-5p* mimics. **P < 0.01, ***P < 0.001, one-way ANOVA. **I***MiR-31-5p* overexpression reduces endogenous H_2_S production in TPC-1 cells. After transfected with *miR-31-5p* mimics, endogenous H_2_S generation in TPC-1 cells was analyzed using AzMc fluorescence probe. *P < 0.05, **P < 0.01, one-way ANOVA. **J** Western blot analysis of CSE, Zeb1, E-cadherin, N-cadherin and MMP-2 protein levels in TPC-1 cells transfected with *miR-31-5p* mimics. Right: Corresponding densitometric quantification visualized as a bubble plot. Both bubble size and color intensity indicate relative protein levels normalized to GAPDH. **K***MiR-31-5p* promotes thyroid cancer cell migration in wound healing assays. Scale bar, 50 μm. Quantification of relative wound closure rate was shown in the right panel. **P < 0.01, one-way ANOVA. **L** Transwell migration/invasion assays with TPC-1 cells following *miR-31-5p* mimics transfection. Scale bar, 50 μm. Quantifications of migrated and invaded cells were shown on the right. *P < 0.05, one-way ANOVA. (M) Stacked bar chart illustrating the percentage of lymph node metastasis (LNM) across four distinct molecular subgroups within the TCGA-THCA cohort. Patients were stratified based on the median expression values of both *CSE* and *miR-31-5p*, resulting in four specific categories. Statistical differences among the groups were evaluated using a Pearson chi square test (*P* < 0.001)

To determine the mechanism governing *CSE* downregulation, we first examined genetic and epigenetic alterations. Analysis of the TCGA cohort revealed that neither copy-number alterations (CNAs) nor promoter methylation differences accounted for *CSE* dysregulation (Fig. S8A, B). Consequently, we focused on post-transcriptional regulation by microRNAs. By intersecting TCGA clinical data (miRNAs negatively correlated with *CSE* but positively with BRAF V600E and TNM stage) with three predictive databases (miRactDB, TargetScan, and miRDB), we identified *miR-31-5p* as the sole candidate satisfying all criteria (Fig. 7A and Fig. S8C).

Sequence analysis predicted a specific binding site for *miR-31-5p* within the *CSE* 3’UTR (1593–1614 nt) (Fig. 7B). Since certain *CSE* splice variants lack this distal 3’UTR, we performed RT-PCR to confirm the presence of this target region. The *miR-31-5p* binding site was detectable in both thyroid cancer cell lines and clinical specimens (Fig. S8D), supporting the potential for regulation. Clinically, *CSE* levels displayed a significant inverse correlation with *miR-31-5p* in the TCGA-THCA cohort (*r* = -0.53, *P* < 0.001; Fig. S8E). Besides, a relationship recapitulated in vitro across a panel of thyroid cell lines (Fig. S8F).

Further analysis of the TCGA dataset showed that *miR-31-5p* was upregulated in PTCs compared to paired normal tissues (Fig. S8G). Besides, *miR-31-5p* expression were increased in BRAF-wildtype tumors and further elevated in the BRAF V600E-mutant group (Fig. 7C). In our local cohort, *miR-31-5p* upregulation was exclusively observed in BRAF V600E-mutant tumors, whereas expression in wildtype cases remained comparable from normal tissues (Fig. 7D). Mechanistically, ectopic expression of BRAF V600E in thyroid cancer cells dramatically induced *miR-31-5p* expression. Importantly, this induction was abrogated by the MEK/ERK inhibitor U0126 (Fig. 7E), confirming that *miR-31-5p* is a downstream effector of the hyperactive MAPK pathway mediating CSE suppression.

To validate CSE as a direct downstream target of *miR-31-5p*, we transfected TPC-1 cells with *miR-31-5p* mimics, which achieved prominent overexpression (Fig. S8H) without compromising cell viability (Fig. S8I). Dual-luciferase reporter assays confirmed that *miR-31-5p* mimics significantly repressed the activity of the wildtype CSE 3’UTR reporter (Fig. 7F), whereas mutation of the predicted binding site abolished this suppressive effect (Fig. 7G). Consistently, ectopic expression of *miR-31-5p* significantly reduced endogenous *CSE* mRNA levels (Fig. 7H) and concomitantly attenuated H_2_S production (Fig. 7I), further verifying the post-transcriptional silencing of *CSE*. Functionally, *miR-31-5p* mimics induced a prominent downregulation of *CSE* and triggered a classic EMT program, characterized by the upregulation of Zeb1, a phenotypic shift from epithelial (E-cadherin) to mesenchymal (N-cadherin) markers, and the concurrent induction of MMP-2 (Fig. 7J). This molecular alteration directly resulted in an aggressive phenotype, as evidenced by significantly enhanced migration and invasion in wound healing and transwell assays (Fig. 7K, L). Further analysis of the TCGA cohort revealed that the *miR-31-5p*^*high*^*/CSE*^*low*^ signature was associated with the highest incidence of lymph node metastasis (62.03%) compared to all other groups (χ^2^ = 30.4, *P* < 0.001; Fig. 7M). In summary, these data demonstrate that *miR-31-5p* acts as a pro-metastatic driver by directly silencing *CSE*, thereby activating the Zeb1/MMP-2 signaling.

### CSE restrains thyroid cancer metastasis by orchestrating intracellular zinc homeostasis

**Fig. 8:**
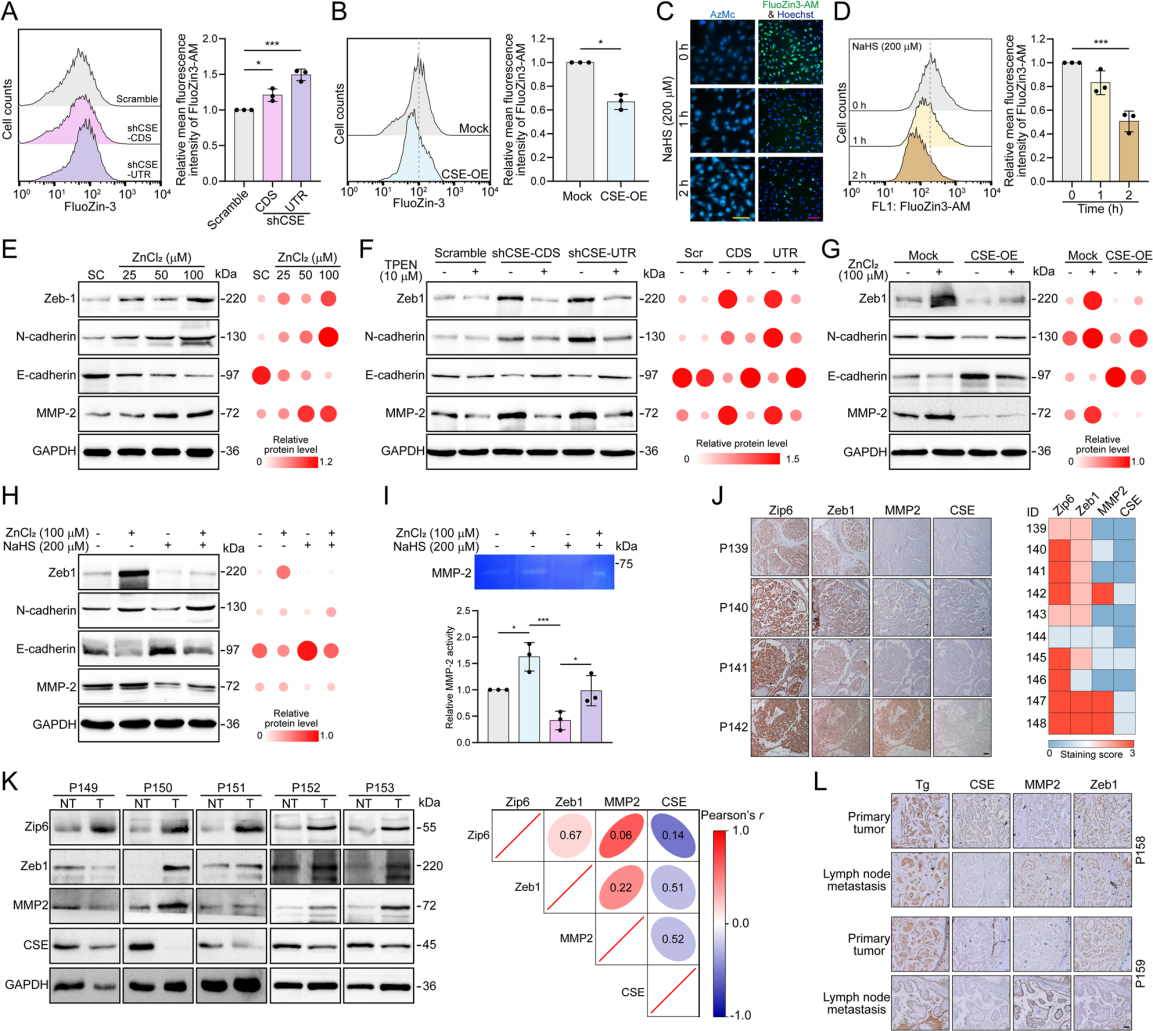
CSE restrains thyroid cancer metastasis by orchestrating intracellular zinc homeostasis. **A** and **B** Flow cytometric analysis of intracellular Zn^2+^ concentration in CSE-knockdown (**A**) and CSE-overexpressed (**B**) cells using FluoZin3-AM. Quantified median fluorescence intensity (MFI) of FluoZin3-AM was shown in the right panels. *P < 0.05, ***P < 0.001, one-way ANOVA or Student’s *t*-test. **C** Dynamic measurement of intracellular H_2_S and Zn²^+^ levels. Representative images showing H_2_S (AzMc probe; blue) and Zn^2+^ (FluoZinc3-AM; green) levels. Scale bar, 100 μm. **D** Flow cytometric analysis of intracellular Zn^2+^ concentration in NaHS-treated KTC-1 cells using FluoZin3-AM. Quantified MFI of FluoZin3-AM was presented in the right panel. ***P < 0.001, one-way ANOVA. **E**-**H** Western blot analysis of the epithelial marker E-cadherin, mesenchymal marker N-cadherin, and zinc-dependent effectors Zeb1 and MMP-2. Quantifications of protein levels were visualized in the adjacent bubble plots. Both bubble size and color intensity represent relative protein levels normalized to GAPDH. **E** ZnCl_2_ increases EMT-related protein expression in KTC-1 cells. **F** Treatment with the zinc chelator TPEN rescues the epithelial phenotype in CSE-silenced cells. **G** Supplementation with ZnCl₂ in CSE-overexpressing cells restores the mesenchymal phenotype. **H** Pharmacological treatment of H₂S attenuates zinc-induced EMT. **I** NaHS reverses ZnCl_2_-induced MMP-2 activation in TPC-1 cells. *P < 0.05, ***P < 0.001, one-way ANOVA. **J** IHC staining of Zip6, Zeb1, MMP-2, and CSE was performed in 10 PTC samples from Jiangyuan Hospital cohort, with four representative images shown. Scale bar, 100 μm. Right panel: heatmap visualization of IHC staining intensity (0 = negative, 1 = weak, 2 = moderate, 3 = strong) for Zip6, Zeb1, MMP-2 and CSE in PTC specimens. **K** Western blot analysis of Zip6, Zeb1, MMP-2 and CSE in 9 paired PTC (T) and adjacent non-tumoral (NT) tissues from Jiangyuan Hospital cohort, with four representative images illustrated. Right panel: correlation matrix visualization of the relationships between Zip6, Zeb1, MMP-2 and CSE expression levels. The numbers in the ellipse represented the *P*-value, Pearson’s correlation test. **L** IHC staining of Zip6, Zeb1, MMP-2 and CSE in 6 matched primary and lymph node metastatic PTC samples from Jiangyuan Hospital cohort. Representative staining images were shown. Scale bar, 100 μm

To mechanically investigated how CSE loss driven metastatic progression, we focused on the potential interplay between H₂S and metal ion metabolism. Given the intrinsic affinity of sulfide for zinc, we hypothesized that CSE might function as a regulator of the intracellular zinc pool. Flow cytometry analysis using the zinc-specific probe FluoZin-3 revealed that CSE silencing triggered a marked accumulation of intracellular zinc, whereas CSE overexpression significantly depleted the zinc pool (Fig. 8A, B). Consistently, treatment with the H₂S donors sustained intracellular H₂S generation and simultaneously reduced Zn²⁺ levels, recapitulating the phenotype of CSE overexpression (Fig. 8C, D, Fig. S9A-C). On the contrary, direct zinc supplementation in TPC-1 cells was sufficient to mimic the CSE-loss phenotype (Fig. 8E), while exhibiting negligible cytotoxicity under both serum-fed and serum-deprived conditions (Fig. S9D).

We next established causality through bidirectional rescue experiments. In CSE-silenced cells, chelation of excess zinc using TPEN, a membrane-permeable zinc chelator, significantly reversed EMT marker expression (Fig. 8F). By contrast, in CSE-overexpressing cells, re-introduction of ZnCl₂ restored EMT-like phenotype (Fig. 8G). Furthermore, the pro-metastatic effect of ZnCl₂ was effectively neutralized by the concurrent administration of the H₂S donor NaHS (Fig. 8H, I). These data provide compelling evidence that CSE suppresses thyroid cancer metastasis primarily by restricting zinc bioavailability *via* H₂S-mediated sequestration.

The role of CSE/H_2_S as a zinc gatekeeper prompted us to investigate the source of the zinc overload. Systematic profiling of the zinc transporter superfamily in the TCGA-THCA dataset revealed that while most *SLC39A* (influx) and *SLC30A* (efflux) members were unchanged, *SLC39A6*, *SLC39A10*, and *SLC30A2* were significantly upregulated in tumors. Notably, elevated *SLC39A6* (encoding Zip6) expression was positively associated with BRAF V600E mutation status (Fig. S9F). This finding demonstrated that Zip6 overexpression might act as the primary driver of zinc influx, creating an oncogenic zinc overload in BRAF-mutated cancers. Importantly, CSE knockdown did not alter the mRNA levels of *SLC39A6* or *SLC39A10*, and *SLC30A2* was undetectable in thyroid cancer cells (Fig. S9G). These results indicate that CSE modulates zinc homeostasis through H₂S-mediated chemical sequestration, rather than by regulating transporter abundance.

To corroborate these dual-hit zinc overload findings in a clinical setting, we first evaluated the in-situ expression patterns of Zip6, CSE, and downstream zinc-dependent effectors within our patient cohort. IHC profiling revealed a distinct signature in these tumors, characterized by high Zip6 expression alongside negligible CSE staining. Consistent with this upstream signature, these tumors generally exhibited concomitant positive expression of Zeb1. While MMP-2 expression was heterogeneous, robust focal positivity was noted in specific cases, including P142, P147, and P148 (Fig. 8J and Fig. S9H). To quantitatively substantiate these observations across a broader cohort, Western blot and Pearson correlation analysis confirmed a robust positive correlation among Zip6, Zeb1 and MMP-2, while revealing that their expression levels are negatively correlated with CSE (Fig. 8K and Fig. S9I). Finally, to provide definitive clinical evidence for the metastatic relevance of this axis, we performed a comparative analysis of paired primary tumors and their corresponding lymph node metastases. While Zeb1 and MMP-2 were elevated in primary tumors compared to normal tissues, their levels were further increased in the lymph node metastases (Fig. 8L and Fig. S9J). This stepwise elevation underscores that the sustained activation of the zinc machinery is a continuous driving force that propels tumor cells from the primary site to metastatic colonization.

## Discussion

Current clinical management of advanced thyroid cancer faces significant challenges. While targeted therapies like BRAF/MEK inhibitors (dabrafenib and trametinib) have shown promising clinical responses, their efficacy focuses primarily on enhancing ^131^I treatment response rather than directly targeting metastatic progression [39]. Additionally, their clinical utility remains limited by inherent and acquired drug resistance, which significantly hinder the improvements in overall survival of BRAF V600E-positive advanced thyroid cancer [40]. These limitations urge the need for developing novel therapeutic strategies to improve outcomes in patients with advanced and metastatic thyroid cancer. The present study identified a novel pathogenic axis wherein BRAF V600E suppresses CSE, resulting in intracellular H_2_S depletion and subsequent metastatic progression.

Our investigation first established CSE downregulation as a critical event connecting BRAF V600E to poor clinical outcomes (Figs. 1, 2 and 3). Unlike CBS and MPST, CSE expression is uniquely silenced in thyroid cancer tissues compared to the high physiological levels observed in normal thyroid [37]. Although the role of H_2_S in cancer remains context-dependent, promoting tumor growth in colorectal cancer while suppressing it in lung cancer [14–16], our gain- and loss-of-function studies definitively establish H_2_S as a potent metastasis suppressor in thyroid cancer (Figs. 4, 5 and 6). Functionally restoring H_2_S effectively inhibited the metastatic potential of aggressive thyroid cancer cells, highlighting the therapeutic value of reactivating this gasotransmitter pathway.

Mechanistically, we deciphered how oncogenic BRAF signaling silences CSE. We identified that *miR-31-5p*, which is directly induced by BRAF V600E, acts as a negative regulator of CSE (Fig. 7). Although the oncogenic role of *miR-31-5p* in thyroid cancer is well-established [41, 42], our study uncovers a novel mechanism by which BRAF-driven overexpression of *miR-31-5p* in thyroid cancers enhances cancer cell motility by suppressing CSE.

A pivotal discovery of this study is the identification of the CSE-H_2_S-Zinc axis as a master regulator of EMT. While physiological zinc acts as a structural cofactor for thousands of proteins, zinc dyshomeostasis is increasingly recognized as a driver of malignancy [43]. Notably, although serum zinc levels are typically depleted in cancer patients, malignant tissues paradoxically exhibit significant zinc accumulation [43]. This discrepancy suggests that whereas physiological zinc intake prevents tumorigenesis, malignant cells develop pathological zinc dependency, particularly for metastatic progression [44]. For advanced thyroid cancer, we attribute this phenotype to a double-hit mechanism, including the upregulation of the zinc importer Zip6 and the concurrent loss of H_2_S-mediated zinc chelation. Zip6 upregulation correlated significantly with BRAF V600E status and EMT markers, suggesting it serves as the primary gateway for oncogenic zinc influx.

We further demonstrated that this accumulated zinc functions not merely as a bystander, but as an essential fuel for the metastatic machinery. Zinc structurally stabilizes and activates key EMT-driving proteins, specifically the zinc-finger transcription factor Zeb1 and the zinc-dependent enzyme MMP-2 [45, 46]. Our data showed that exogenous zinc supplementation was sufficient to trigger EMT and mimic the CSE-loss phenotype, whereas H_2_S donors could chelate excess zinc to inactivate Zeb1 and MMP-2 (Fig. 8). These findings align with recent report in pancreatic cancer where zinc accumulation drives Zeb1 activation cells [47], confirming that H_2_S deficiency creates a favorable metallo-environment for EMT execution.

Our findings also offer a molecular explanation for the clinical heterogeneity observed in BRAF-mutated DTC. While the coexistence of BRAF V600E and *TERTp* mutations is unequivocally linked to the aggressive outcomes, this specific genotype accounts for only a small fraction of cases. Indeed, in our own cohort of 1,728 fine-needle aspiration (FNA) samples, *TERTp* mutations were identified in only 1% of cases (17/1,728). In sharp contrast, postoperative pathology from the same period revealed a lymph node metastasis rate of 46.3% (252/544). This leaves the metastatic drivers in the prevailing *TERTp*-wildtype population largely elusive. Consequently, our findings position CSE deficiency as a potential feature that fuels metastasis in this broader patient subgroup.

From a therapeutic perspective, our findings provide a rationale for H_2_S-based interventions. While H_2_S donors have shown variable effects in other cancers [48], our previous and current data consistently support the anti-tumor efficacy of H_2_S donors, including DATS, GYY4137 and NaHS in thyroid cancer [17, 18]. By serving as an endogenous zinc chelator, H₂S offers a strategy to target the intracellular zinc accumulation characterizing BRAF-mutated tumors. Given the clinical progression of H₂S -releasing agents like SG1002 and ATB-346 for other pathologies [49], repurposing these agents or developing thyroid-targeted H₂S might offer a complementary strategy to help address the challenges associated with current therapies. More broadly, strategies that directly target zinc accumulation within tumor tissues may represent another promising therapeutic strategy against thyroid cancer metastasis.

Finally, our study has limitations that warrant further investigation. First, although the tail vein injection model in NCG mice effectively mimics thyroid cancer lung metastasis, it artificially bypasses the initial intravasation step of the metastatic cascade. Consequently, validating our findings in immunocompetent and genetically engineered spontaneous thyroid cancer models represents a promising future direction. Besides, although we have identified the H₂S-mediated sequestration of zinc as a primary mechanism inactivating Zeb1 and MMP-2, H₂S also functions through protein S-sulfhydration [50]. Whether H₂S directly modifies the cysteine residues of these zinc-dependent effectors to alter their conformation or activity remains to be explored. Finally, although we established a link between BRAF V600E and Zip6 upregulation, the precise transcriptional machinery bridging the MAPK pathway to *SLC39A6* requires further investigation.

## Conclusions

Collectively, our study reveals the CSE/H_2_S system as a critical but previously unrecognized guardian against thyroid cancer progression. We demonstrate that BRAF V600E mutation drives *miR-31-5p*-mediated suppression of CSE expression, leading to H_2_S deficiency that subsequently disrupts zinc homeostasis *via* impaired H_2_S-medieated Zn^2+^ chelation, ultimately resulting in the activation of Zeb1/MMP-2-dependent metastatic pathways. Our findings underscore the therapeutic potential of targeting the H_2_S-zinc axis in thyroid cancer. Specifically, H_2_S supplementation and zinc deplete can effectively inhibit metastatic progression. These approaches may provide novel treatment strategies for aggressive thyroid cancer, particularly in BRAF V600E-mutant cases resistant to current therapies.

## Supplementary Information

Supplementary Material 1.

Supplementary Material 2.

Supplementary Material 3.

Supplementary Material 4.

Supplementary Material 5.

## Data Availability

No datasets were generated or analysed during the current study.

## Abbreviations

DTC: Differentiated thyroid cancer
PTC: Papillary thyroid carcinoma
FTC: Follicular thyroid cancer
PDTC: Poorly differentiated thyroid cancer
ATC: Anaplastic thyroid cancer
TNBC: Triple-negative breast cancer
AJCC: American Joint Committee on Cancer
LNM: Lymph node metastasis
CSE: Cystathionine γ lyase
CBS: Cystathionine-β-synthase
*MPST*: 3-mercaptopyruvate sulfurtransferase
Tg: Thyroglobulin
TTF1: Thyroid transcription factor-1
EMT: Epithelial-mesenchymal transition
EMT-TFs: EMT-related transcription factors
BRAF: B-Raf proto-oncogene, serine/threonine kinase
MAPK: Mitogen-activated protein kinase
SEER: Surveillance, Epidemiology, and End Results
DSS: Disease-specific survival
CDS: Coding sequences
3’ UTR: 3’ untranslated regions
SCR: Scrambled
Luc: Luciferase
TCGA: The Cancer Genome Atlas
GEO: Gene Expression Omnibus
HPA: Human Protein Atlas
GO: Gene Ontology
KEGG: Kyoto Encyclopedia of Genes and Genomes
GSEA: Gene Set Enrichment Analysis
PCA: Principal component analysis
FFPE: Formalin-fixed paraffin-embedded
RT-PCR: Reverse transcriptase PCR
qPCR: Quantitative real-time PCR
H&E: Hematoxylin-eosin
IHC: Immunohistochemistry
IF: Immunofluorescence
ROC: Receiver operating characteristic
AUC: Area under the curve
MMPs: Matrix metalloproteinases
ECM: Extracellular matrix
MIP: Maximum intensity projection
CNA: Copy-number alteration
DATS: Diallyl trisulfide
NaHS: Sodium hydrosulfide
