# Supplementary material for: Loss of CSE couples with BRAF V600E to fuel thyroid cancer metastatic progression through zinc-dependent activation of the EMT machinery
Source: J Exp Clin Cancer Res. 2026 Jun 10;45:178. doi: 10.1186/s13046-026-03752-0 (PMC13474713; doi:10.1186/s13046-026-03752-0)
Supplement: Supplementary file 1 [file 13046_2026_3752_MOESM1_ESM.docx]

**Supplementary Materials**

**Loss of CSE couples with BRAF V600E to fuel thyroid cancer metastatic progression through zinc-dependent activation of the EMT machinery**

**Supplementary Figures contents:**

**Figure S1.** CSE is abnormally repressed in thyroid cancers.

**Figure S2.** CSE promotes thyroid differentiation and functional radioiodine uptake in PTC cells.

**Figure S3.** BRAF V600E mutation acts as an upstream suppressor of CSE expression.

**Figure S4.** Loss of CSE is associated with capsular invasion and spatial EMT activation in PTC.

**Figure S5.** The inhibitory effect of CSE on thyroid cancer metastasis depends on its capacity to catalyze the release of endogenous H₂S.

**Figure S6.** Exogenous H₂S donors effectively inhibit thyroid cancer cell migration and invasion.

**Figure S7.** CSE-mediated suppression of thyroid cancer lung metastasis in immunodeficient mouse models.

**Figure S8.** *MiR-31-5p* mediates BRAF V600E-driven CSE suppression and promotes thyroid cancer cell metastasis.

**Figure S9.** Zinc dyshomeostasis and specific zinc transporter alterations critically drive thyroid cancer invasion and metastasis.

**Figure S1. CSE is abnormally repressed in thyroid cancers.** (A and B) Expression profiles of *CBS* (A) and *MPST* (B) in PTC tumoral tissues (T) *versus* non-tumoral (NT) tissues from the TCGA and GTEx databases. (C) RMA-normalized mRNA expression levels of *CSE* in matched tumoral/non-tumoral thyroid tissue pairs from GSE35570, GSE60542 and GSE33630 datasets. *P* < 0.0001, two-tailed Student’s paired *t*-test. (D) RPKM-normalized expression values of *CSE* across normal organs from Human BodyMap 2.0 database (<https://www.ensembl.info/2011/05/24/human-bodymap-2-0-data-from-illumina/>). (E) Receiver Operating Characteristic (ROC) analysis of *CSE* expression for discriminating benign and malignant thyroid nodule. (F) Representative immunohistochemical (IHC) staining images of CSE in PTC and normal thyroid samples from Human Protein Atlas (HPA) database. The staining intensity of CSE was shown in the right panel. (G) Representative immunofluorescence (IF) staining images of CSE in PTC specimens from Jiangyuan Hospital cohort. Scale bar, 100 μm. Nuclei were counterstained with Hoechst.

**Figure S2. CSE promotes thyroid differentiation and functional radioiodine uptake in PTC cells.** (A) *CSE* mRNA expression *versus* thyroid cancer differentiation score in TCGA-THCA (Pearson correlation test). (B) Western blot analysis evaluating the protein expression levels of iodine-metabolizing genes (TTF-1, Pax8, NIS, and TPO), cancer stem cell markers (CD44 and CD133), and stem cell transcription factors (OCT4A, Nanog, and SOX2). Assays were performed in TPC-1 cells following CSE knockdown (shCSE-CDS and shCSE-UTR; left panel) and in KTC-1 cells following CSE overexpression (CSE-OE; right panel). GAPDH and Tubulin served as the loading controls, respectively. (C) Functional radioiodine uptake assays evaluated over a 120-minute time course in CSE-silenced TPC-1 cells (top) and CSE-overexpressing KTC-1 cells (bottom). ****P* < 0.001, two-way ANOVA; n.s., not significant.

**Figure S3. BRAF V600E mutation inhibits CSE expression.** (A) Western blot analysis of CSE protein expression in human normal thyroid follicular epithelial cells (Nthy-ori-3.1); PTC-derived cell lines (TPC-1, KTC-1, BCPAP); ATC-derived cell lines (8505C, BHT101, Cal-62, C643). BRAF mutation status (top) and relative CSE expression (bottom) were indicated for each cell line. In the adjacent bubble plot, both the size and color intensity of each bubble correspond to the relative fold-change of protein expression normalized to GAPDH. (B) mRNA expression of *CSE* in a BRAF V600E-induced PTC model (FVB/N strain). (C) *CSE* mRNA expression in BRAF V600E transgenic mice. *CSE* mRNA levels in thyroid tissues from wildtype (WT) and BRAF V600E transgenic mice after tamoxifen treatment. **P* < 0.05, Student’s *t*-test. Expression profiling of CSE in anaplastic thyroid cancer stratified by BRAF mutation status. (D-E) Analysis of *CSE* mRNA levels between BRAF wild-type (WT) and BRAF V600E-mutated ATC samples using transcriptomic data from the TCGA-MSKCC cohort (D, WT n = 9, V600E n = 11) and the TCGA-GATCI cohort (E, WT n = 16, V600E n = 5). (F) Relative CSE protein expression in normal thyroid tissues (n = 119) and ATC samples stratified by BRAF status (non-V600E n = 36, V600E n = 15) based on proteomic data from the HRA007805 dataset. **P* < 0.05 and ***P* < 0.01, Kruskal‑Wallis H; where n.s. indicates not significant, Mann‑Whitney U.

**Figure S4. Loss of CSE is associated with capsular invasion and spatial EMT activation in PTC.** (A) *CSE* mRNA expression in PTC tissues with or without extrathyroidal extension (GSE29265 dataset). **P* < 0.05, Student’s *t*-test. (B) Side-by-side comparison of the EMT signature score and raw *CSE* expression at single-spot resolution across the tissue section.

**Figure S5. The inhibitory effect of CSE on thyroid cancer metastasis depends on its capacity to catalyze the release of H_2_S.** (A) Proliferation analysis in CSE-knockdown TPC-1 cells. N.S., no significance, two-way ANOVA. Insert: Western blot confirmation of CSE knockdown efficiency in TPC-1 cells using lentiviral shRNA vectors. (B) Cell viability analysis in TPC-1 cells following PAG treatment (0-8 mM, 48 h). n.s., one-way ANOVA. (C) Inhibition of endogenous H_2_S production by PAG in TPC-1 cells. Cells were pretreated with 1 mM of PAG, a CSE inhibitor, for 48 h and then stained with 10 μM of AzMc. Intracellular H_2_S levels were examined by fluorescence spectrophotometry. ****P* < 0.001, Student’s *t*-test. (D) PAG (1 mM) promotes TPC-1 cell migration in wound healing assays. Scale bar, 50 μm. **P* < 0.05, Student’s *t*-test. (E) PAG (1 mM) enhances migration and invasion of TPC-1 cells in transwell assays. Scale bar, 50 μm. **P* < 0.05, Student’s *t*-test. (F) PAG treatment (1 mM) alters EMT and invasion-related protein expression. The adjacent bubble plot displays Tubulin-normalized protein fold changes through both bubble size and color. (G) PAG (1 mM) increases MMP-2 activity of TPC-1 cells in gelatin zymography assay. **P* < 0.05, Student’s *t*-test. (H) Proliferation of KTC-1 cells with CSE overexpression *versus* empty vector control (mock). N.S., two-way ANOVA. Insert: Western blot confirmation of CSE overexpression in KTC-1 cells using lentivirus. (I) Two-dimension interaction maps detailing the binding modes of L-Cystathionine with WT, E339A, and D187A/T189A CSE variants.

**Figure S6. Exogenous H_2_S donors inhibit thyroid cancer cell metastasis.** (A) Cell viability of KTC-1 cells following treatment with exogenous H₂S donors. Cells were treated with increasing dosages of DATS, GYY4137 or NaHS for 24 h, and then cell viability was measured using MTT assay. ***P* < 0.01 *versus* control group, one-way ANOVA. N.S., no significance. SC, solvent control. (B and C) DATS (B) and GYY4137 (C) inhibit KTC-1 cell migration in wound healing assays. Scale bar, 50 μm. Quantifications of relative wound closure rates were shown in the right panel. ***P* < 0.01 *versus* solvent control group, Student’s *t*-test. (D and E) DATS (D) and GYY4137 (E) inhibit the migration and invasion of KTC-1 cells in transwell assays. Scale bar, 50 μm. Quantitative analysis of relative migration and invasion rates was shown in the right panels. **P* < 0.05, ***P* < 0.01 *versus* solvent control group, Student’s *t*-test.

**Figure S7. CSE-mediated suppression of thyroid cancer metastasis in mouse models.** (A) Western blot analysis of integrin α2 and integrin β1 in Nthy-ori-3.1 and thyroid cancer cell lines. (B) Representative PET/MR images of thyroid cancer lung metastases in NCG mice following ^68^Ga-NOTA-RGD administration. Non-fused PET, MR images at the same axial level and maximum intensity projection (MIP) views were shown. (C) NCG mice were intravenously injected with 7.5×10^5^ of thyroid cancer cells to establish metastatic lesions. Left panel: representative coronal synchronous SPECT/CT image at 60 min post 3.7  MBq of Na^131^I intravenous administration. Right panel: representative coronal PET, synchronous PET/MR and MIP images at 60 min post 3.7  MBq of ^18^F-FDG intravenous administration. (D) Longitudinal body weight monitoring of NCG mice intravenously injected with scramble control or CSE-knockdown TPC-1 cells. N.S., two-way repeated measures ANOVA. (E) Longitudinal body weight monitoring of NCG mice following intravenous injection of mock-transfected or CSE-overexpressed KTC-1 cells. N.S., two-way repeated measures ANOVA. (F) Histopathological and immunohistochemical analyses of lung metastases. Representative H&E and IHC staining of lung sections from NCG mice euthanized 4 weeks post-intravenous injection with mock-transfected or CSE-overexpressed KTC-1 cells. IHC staining for CSE, Tg, TTF1 and Ki67 was shown. Scale bar, 50 μm. (G) IVIS images of lung metastases in NCG mice following intravenous injection of 7.5×10^5^ mock-transfected or CSE-overexpressed Cal-62/C643 cells. (H) Representative H&E and IHC staining of lung sections from NCG mice euthanized 4 weeks post-intravenous injection of mock-transfected or CSE-overexpressed KTC-1 cells. IHC staining for CSE, Tg, TTF1 and Ki67 was shown. Scale bar, 100 μm.

**Figure S8. *MicroRNA-31-5p* mediates BRAF V600E-driven CSE suppression and promotes thyroid cancer cell metastasis.** (A) Spectrum of genetic alterations in *CSE* (TCGA-THCA dataset). (B) Comparative analysis of CpG island methylation in *CSE* promoter between PTC (T) and non-tumoral (NT) tissues from TCGA-THCA dataset. (C) Venn diagram analysis of *CSE*-targeting miRNAs predicted by three bioinformatic tools. (D) Validation of *miR-31-5p* binding site in *CSE* 3'UTR across thyroid cancer tissues and cell lines. PCR amplification of *CSE* 3'UTR region and corresponding Sanger sequencing chromatograms were shown. BRAF mutation status was indicated above for each cell line. (E) Correlation between *CSE* and *miR-31-5p* levels in PTC tissues (TCGA-THCA). *P* < 0.001, Pearson correlation test. (F) Differential expression of CSE protein and *miR-31-5p* in thyroid cell lines. Bubble plot summary of the relative levels of CSE protein and *miR-31-5p*. (G) *MiR-31-5p* expression levels in matched thyroid tumoral/non-tumoral pairs (n=59) from TCGA-THCA dataset. ****P* < 0.001, two-tailed Student’s paired *t*-test. (H) QPCR analysis of *miR-31-5p* levels in TPC-1 cells transfected with *miR-31-5p* mimics. ****P* < 0.001, one-way ANOVA. (I) Effects of *miR-31-5p* on TPC-1 cell viability. **P* < 0.05, ***P* < 0.01, ****P* < 0.001, one-way ANOVA.

**Figure S9. Zinc dyshomeostasis critically drives thyroid cancer invasion and metastasis.** (A) Dynamic measurement of intracellular H_2_S and Zn²^+^ levels after DATS and GYY4137 treatment. Representative images showing H_2_S (AzMc probe; blue) and Zn^2+^ (FluoZinc3-AM; green) levels. Scale bar, 100 μm. (B and C) Flow cytometric analysis of intracellular Zn^2+^ concentration in DATS (B) and GYY4137 (C) treated KTC-1 cells using FluoZin3-AM. Quantified median fluorescence intensity (MFI) of FluoZin3-AM was presented in the right panel. **P* < 0.05, ***P* < 0.01, one-way ANOVA. (D) Effects of ZnCl_2_ on TPC-1 cell viability under serum-containing and serum-free culture conditions. N.S., **P* < 0.05, ***P* < 0.01 *versus* solvent control group, one-way ANOVA. (E) Cell viability of TPC-1 cells treated with NaHS (200 μM) in the presence or absence of ZnCl_2_ (100 μM) for 24 h, as determined by MTT assay. N.S., *versus* control group, one-way ANOVA. (F) Heatmap analysis of zinc transporter families in thyroid tissues stratified by BRAF V600E mutation status (TCGA-THCA). (G) QPCR analysis of *SLC39A6*, *SLC39A10* and *SLC30A2* mRNA levels in CSE-knockdown TPC-1 cells. N.S., one-way ANOVA. (H) IHC staining of Zip6, Zeb1, MMP-2 and CSE in six additional PTC samples (related to Fig. 7J). Scale bar, 100 μm. (C) Western blot analysis of Zip6, Zeb1, MMP-2, and CSE protein expression in additional five paired PTC (T) and adjacent non-tumoral (NT) tissues (related to Fig. 7K). (D) IHC staining of Zip6, Zeb1, MMP-2 and CSE in four additional matched primary and lymph node metastatic PTC samples (related to Fig. 7L). Scale bar, 100 μm.
