# Supplementary material for: Loss of CSE couples with BRAF V600E to fuel thyroid cancer metastatic progression through zinc-dependent activation of the EMT machinery
Source: J Exp Clin Cancer Res. 2026 Jun 10;45:178. doi: 10.1186/s13046-026-03752-0 (PMC13474713; doi:10.1186/s13046-026-03752-0)
Supplement: Supplementary file 2 [file 13046_2026_3752_MOESM2_ESM.docx]

**Supplementary Materials**

**Loss of CSE couples with BRAF V600E to fuel thyroid cancer metastatic progression through zinc-dependent activation of the EMT machinery**

**Table of Contents** **(results-related)**

**Table S1.** Correlation between CSE expression and clinicopathological features in PTC from the TCGA database.

**Table S2.** Multivariate logistic regression analysis of independent predictors for lymph node metastasis in thyroid carcinoma.

**Table S1.** Correlation between *CSE* expression and clinicopathological features in PTC from TCGA database (TCGA, Cell 2014) [1].

| **Clinicopathologic Parameters** | **Number of specimens** | ***CSE* expression**^a^ | | ***P* value**^b^ |
| --- | --- | --- | --- | --- |
|  |  | **Low** | **High** |  |
| **Gender** |  |  |  | 0.916 |
| Female | 331 (72.9%) | 166 (73.1%) | 165 (72.7%) |  |
| Male | 123 (27.1%) | 61 (26.9%) | 62 (27.3%) |  |
| **Age at diagnosis** |  |  |  | 0.547 |
| <55 | 308 (67.8%) | 157 (69.2%) | 151 (66.5%) |  |
| ≥55 | 146 (32.2%) | 70 (30.8%) | 76 (33.5%) |  |
| **pT status** |  |  |  | 0.202 |
| Tx | 2 (0.4%) | 2 (0.9%) | 0 (0.0%) |  |
| T1-T2 | 283 (62.3%) | 135 (59.5%) | 148 (65.2%) |  |
| T3-T4 | 169 (37.2%) | 90 (39.6%) | 79 (34.8%) |  |
| **pN status** |  |  |  | **< 0.001** |
| Nx | 45 (9.9%) | 15 (6.6%) | 30 (13.2%) |  |
| N0 | 208 (45.8%) | 86 (37.9%) | 122 (53.7%) |  |
| N1 | 201 (44.3%) | 126 (55.5%) | 75 (33.0%) |  |
| **pM status^c^** |  |  |  | 0.422 |
| Mx | 242 (53.4%) | 121 (53.3%) | 121 (53.5%) |  |
| M0 | 8 (1.8%) | 6 (2.6%) | 2 (0.9%) |  |
| M1 | 203 (44.8%) | 100 (44.1%) | 103 (45.6%) |  |
| **Stage at diagnosis^d^** |  |  |  | 0.055 |
| I-II | 309 (68.4%) | 145 (64.2%) | 164 (72.6%) |  |
| III-IV | 143 (31.6%) | 81 (35.8%) | 62 (27.4%) |  |
| **Overall survival status^e^** |  |  |  | > 0.999 |
| Living | 372 (97.4%) | 184 (97.4%) | 188 (97.4%) |  |
| Deceased | 10 (2.6%) | 5 (2.6%) | 5 (2.6%) |  |
| **Disease-free status^f^** |  |  |  | 0.159 |
| Disease Free | 372 (93.7%) | 184 (92.0%) | 188 (95.4%) |  |
| Recurred/Progressed | 25 (6.3%) | 16 (8.0%) | 9 (4.6%) |  |
| **Histology subtype** |  |  |  | **< 0.001** |
| Papillary, classical | 312 (68.7%) | 172 (75.8%) | 140 (61.7%) |  |
| Follicular variant | 99 (21.8%) | 30 (13.2%) | 69 (30.4%) |  |
| Tall-cell variant | 34 (7.5%) | 20 (8.8%) | 14 (6.2%) |  |
| Other | 9 (2.0%) | 5 (2.2%) | 4 (1.8%) |  |

^a^The median mRNA expression (RNA Seq V2 RSEM) of *CSE* is 55.56. A total of 454 samples were divided into two groups based on the median. The mRNA expression level ≥ 55.56 was considered as high; < 55.56 was considered as low.

^b^Pearson’s chi-squared test was used for comparison of gender, age, pN, stage, disease-free status, thyroid differentiation score, *BRAF*, *RAS* and *TERTp* status. Fisher's exact test was used for comparison of pT, pM, overall survival status and histology subtype.

^c^Partial data not available; analysis of pM status was available for 453 cases.

^d^Partial data not available; analysis of Stage at diagnosis was available for 452 cases.

^e^Partial data not available; analysis of Overall survival status was available for 382 cases.

^f^Partial data not available; analysis of Disease-free status was available for 397 cases.

pT, American Joint Committee on Cancer Tumor Stage Code; pN, American Joint Committee on Cancer Lymph Node Stage Code; pM, American Joint Committee on Cancer Metastasis Stage Code.

**Table S2**. Multivariate logistic regression analysis of independent predictors for lymph node metastasis in thyroid carcinoma (TCGA, Cell 2014)[1].

| Variables | Odds Ratio, OR | 95% CI | *P*-value |
| --- | --- | --- | --- |
| *CSE* Expression |  |  |  |
| *CSE*-high | 1.00 (Ref.) |  |  |
| *CSE*-low | 1.91 | 1.28 - 2.85 | **0.001** |
| BRAF Status |  |  |  |
| Wild type | 1.00 (Ref.) |  |  |
| Mutant | 1.95 | 1.29 - 2.94 | **0.001** |
| Gender |  |  |  |
| Female | 1.00 (Ref.) |  |  |
| Male | 1.56 | 1.01 - 2.41 | **0.046** |
| Age |  |  |  |
| <55 | 1.00 (Ref.) |  |  |
| ≥55 | 0.53 | 0.35 - 0.81 | **0.003** |
| T stage |  |  |  |
| T1-T2 | 1.00 (Ref.) |  |  |
| T3-T4 | 2.53 | 1.70 - 3.80 | **< 0.001** |

**Reference:**

1.	Cancer Genome Atlas Research, N., *Integrated genomic characterization of papillary thyroid carcinoma.* Cell, 2014. **159**(3): p. 676-90. doi:10.1016/j.cell.2014.09.050.
