# Supplementary material for: Loss of CSE couples with BRAF V600E to fuel thyroid cancer metastatic progression through zinc-dependent activation of the EMT machinery
Source: J Exp Clin Cancer Res. 2026 Jun 10;45:178. doi: 10.1186/s13046-026-03752-0 (PMC13474713; doi:10.1186/s13046-026-03752-0)
Supplement: Supplementary file 3 [file 13046_2026_3752_MOESM3_ESM.docx]

**Supplementary Materials**

**Loss of CSE couples with BRAF V600E to fuel thyroid cancer metastatic progression through zinc-dependent activation of the EMT machinery**

**Table of Contents (experimental reagents)**

**Table S3.** List of primary antibodies used in this study.

**Table S4.** List of primer sequences used for RT-PCR and qPCR analysis.

**Table S3.** The primary antibodies used in this research.

| **Primary antibody** | **Epitope/Specificity** | **Host species** | **Type** | **Working dilution** | **Source** | **Cat. number** |
| --- | --- | --- | --- | --- | --- | --- |
| anti-ERK | Total p44/42 MAP kinase (Erk1/Erk2) protein | Rabbit | IgG | WB: 1:1000 | Cell Signaling Technology | 4695 |
| anti-phospho-ERK (Thr202/Tyr204) | Thr202 and Tyr204 of Erk1 (Thr185 and Tyr187 of Erk2) | Rabbit | IgG | WB: 1:2000 | Cell Signaling Technology | 4370 |
| anti-CSE | CSE | Mouse | IgG1 | WB: 1:1000  IHC: 1:200 | Proteintech Group | 60234-1-Ig |
| anti-MMP-2 | MMP2 | Rabbit | IgG | WB: 1:500  IHC: 1:100 | Proteintech Group | 10373-2-AP |
| anti-Zeb1 | Zeb1 | Mouse | IgG1 | WB: 1:1000  IHC: 1:200 | Proteintech Group | 66279-1-Ig |
| anti-Thyroglobulin | Thyroglobulin (Tg) | Rabbit | IgG | IHC: 1:100 | Proteintech Group | 21714-1-AP |
| anti-TTF1 | TTF1 | Mouse | IgG3 | IHC: 1:50 | Proteintech Group | 66034-1-Ig |
| anti-Zip6 | Zip6 | Rabbit | IgG | WB: 1:1000  IHC: 1:200 | Proteintech Group | 14236-1-AP |
| anti-BRAF V600E | BRAF V600E | Mouse | IgG2 | IHC: 1:100 | Sangon Biotech | D195496 |
| anti-N-cadherin | N-cadherin | Rabbit | IgG | WB: 1:1000 | Beyotime Biotechnology | AF5237 |
| anti-E-cadherin | E-cadherin | Mouse | IgG1 | WB: 1:500 | Beyotime Biotechnology | AF0138 |
| anti-Ki67 | Ki67 | Rabbit | IgG | IHC: 1:100 | Beyotime Biotechnology | AF1738 |
| anti-GAPDH | GAPDH | Mouse | IgG1 | WB: 1:2000 | Beyotime Biotechnology | AF0006 |
| anti-Tubulin | Tubulin | Mouse | IgG1 | WB: 1:2000 | Beyotime Biotechnology | AF2827 |

**Table S4.** The nucleotide sequences for both RT-PCR and qPCR primers used in this study (5' to 3').

| **RT-PCR** |  |
| --- | --- |
| *miR-31-5p* Stem-loop RT primer | GTCGTATCCAGTGCAGGGTCCGAGGTATTCGCACTGGATACGACAGCTATG |
| *CSE-F* | AGCCTTCATAATAGACTTCG |
| *CSE-R* | CACTGACGCTTCACCAAC |
| *CBS-F* | ATCGGCTACGACTTCATCCC |
| *CBS-R* | CCCAAGCGTCACCATTCC |
| *MPST-F* | CCCTACGACCACATGCTGC |
| *MPST-R* | CAGGTTCAATGCCGTCTCG |
| *MMP-2-F* | TTTGACGGTAAGGACGGACTC |
| *MMP-2-R* | CCTGGAAGCGGAATGGAA |
| *CSE-3'UTR-F* | GGTACGTCCTTGTAGTCC |
| *CSE-3'UTR-R* | ATAGTCACGGCTTCTGTT |
| *Actin-F* | GCCGGGACCTGACTGACTAC |
| *Actin-R* | CGGATGTCCACGTCACACTT |
| **Q-PCR** |  |
| *CSE-F* | CCTCTCATCCACAGCATGAGTTGGT |
| *CSE-R* | CTCGGCCAGAGTAAATAG |
| *MiR-31-5p-F* | GCAGGCAAGATGCTGGC |
| *MiR-31-5p-R* | GTGCAGGGTCCGAGGT |
| *U6-F* | CTCGCTTCGGCAGCACA |
| *U6-R* | AACGCTTCACGAATTTGCGT |
| *SLC39A6-F* | AAAGGCTGGCATGACCGTTA |
| *SLC39A6-R* | CGGCTACATCCATGGTCACT |
| *SLC39A10-F* | ATTCCTCGGACGATCCATGTG |
| *SLC39A10-R* | TCCACGATGCTGTCTGTGAA |
| *SLC30A2-F* | GACCATGAACTTTGGCTGGC |
| *SLC30A2-R* | GGTGAAGGGTCAACCCCATT |
| *GAPDH-F* | TCAAGAAGGTGGTGAAGCA |
| *GAPDH-R* | AAAGGTGGAGGAGTGGGT |
